# Dyke demolition led to a sharp decline in waterbird diversity due to habitat quality reduction: A case study of Dongting Lake, China

**DOI:** 10.1002/ece3.8782

**Published:** 2022-03-31

**Authors:** Feng Zhu, Yeai Zou, Pingyang Zhang, Siqi Zhang, Xinsheng Chen, Feng Li, Zhengmiao Deng, Hong Zhang, Zhibing Yu, Xiaoyong Zhu, Yonghong Xie, Dongsheng Zou

**Affiliations:** ^1^ 12575 College of Resources and Environment Hunan Agricultural University Changsha China; ^2^ Hunan Key Laboratory of Remote Sensing Monitoring of Ecological Environment in Dongting Lake area Hunan Natural Resources Affairs Center Changsha China; ^3^ Key Laboratory of Agro‐ecological Processes in Subtropical Regions Chinese Academy of Sciences Changsha China; ^4^ Dongting Lake Station for Wetland Ecosystem Research Institute of Subtropical Agriculture Chinese Academy of Sciences Changsha China; ^5^ University of Chinese Academy of Sciences Beijing China; ^6^ Hunan Provincial Key Laboratory of Rural Ecosystem Health in Dongting Lake Area Changsha China; ^7^ School of Resources and Environmental Engineering Anhui University Hefei Anhui Province China; ^8^ Administrative Bureau of Hunan East Dongting Lake National Nature Reserve Yueyang China

**Keywords:** biodiversity, Dongting Lake, Dyke demolition, habitat change, waterbird, wetlands

## Abstract

Dongting Lake, an important wintering habitat for migratory waterbirds in the East Asian–Australasian Flyway, has suffered serious degradation in recent decades. To restore habitats for biodiversity conservation and flood control, 459 dykes were demolished and 14 were preserved in 2017. However, the direct impact of dyke demolition on wintering waterbirds was not comprehensively assessed. In this study, based on annual waterbird census and habitat data (2013/14–2020/21), we compared the differences in habitat areas and species composition of waterbirds in the dyke‐demolished and preserved areas, and explored whether habitat changes caused by the dyke demolition were responsible for the changes in the number of species and percentages of waterbird individuals. The results indicate that the areas of water (including shallow water) and mudflat habitats significantly decreased, but the vegetation area significantly increased in the dyke‐demolished areas. The species numbers and percentages of waterbird individuals at the community and foraging guilds levels, and the percentages of nine species, were higher in the dyke‐preserved areas than those in the dyke‐demolished areas. Changes in the numbers of species and percentages of individuals of fish eaters, insectivores, and omnivores positively correlated with drastic changes in the percentages of water habitats (including shallow water) after dyke demolition. Effective measures should be carried out to restore hydrological regimes, providing waterbirds sufficient suitable habitats with different water depths. These findings improve our understanding of the influence of dyke demolition on waterbirds and provide insights for wetland management and waterbird conservation.

## Graphical abstract

Dyke demolition led to a sharp decline in waterbird diversity at the community, foraging guilds, and species levels. Habitat changes caused by dyke demolition were responsible for the changes in waterbird diversity.

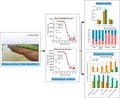

## 1 INTRODUCTION

Wetlands provide important ecological services (e.g., biodiversity maintenance; Hansson et al., 2005; Zedler & Kercher, 2005), but can suffer serious loss and degradation as the result of human activities (Dang et al., 2020; Hu et al., 2017). These activities often alter the hydrological regime of wetlands and thus pose a threat to their biodiversity (Foti et al., 2013; Jones et al., 2021; Palmer & Ruhi, 2019). Therefore, the impact of anthropogenic modifications of wetland hydrological regimes on biodiversity has important research implications.

The Yangtze River floodplain is subject to monsoonal flooding and seasonal hydrological fluctuations (inundation and reduction of water levels in summer and autumn, respectively; Fang et al., 2006; Liu et al., 2020). This unique hydrological cycle creates many extensive and ephemeral artificial and natural wetlands (Xia et al., 2017), which support more than one million wintering waterbirds along the East Asian–Australasian Flyway, including populations of several globally threatened species (Cao et al., 2010; Wang et al., 2017). Unfortunately, since the 1970s, wetland areas along the middle reaches of the Yangtze River basin have been severely decreased (Cui et al., 2013). Xie et al. (2017) reported that from 1975 to 2015, an area of 2132.3 ± 219.6 km^2^ was lost from the total area of all lakes along the middle and lower reaches of the Yangtze River, with Dongting Lake experiencing the most obvious decline (855.1 ± 131.8 km^2^). Similarly, the health of wetland ecosystems is also declining (Sun et al., 2017). Human activities and excessive reclamation are the main driving factors leading to wetland degradation (Du et al., 2011; Hou, Feng, et al., 2020). Despite these decreases, the middle and lower reaches of the Yangtze River basin remain the most important wintering habitat for migratory waterbirds in China. Waterbird communities are key indicators for assessing wetland health and ecosystem services because they respond to variations in wetland environmental factors (Kingsford, 1999; Ogden et al., 2014; Williamson et al., 2013). Therefore, environmental shifts in wetland ecosystems can be reflected by changes in waterbird diversity.

Dongting Lake, located on the middle and lower reaches of the Yangtze River, is the second‐largest freshwater lake in China and one of 200 priority ecoregions for global conservation (Olson & Dinerstein, 2002). The Yangtze River Plain has been intensively exploited for the development of the social economy, including the construction of the Three Gorges Dam, reclamation, and sand mining. After the operation of the Three Gorges Dam, hydrological regimes inevitably changed (in particular, the unusually early water recession), reducing habitat suitability for wintering waterbirds in Dongting Lake (Sun et al., 2012; Wu et al., 2020). Earthen dykes were historically constructed in the Dongting Lake area as part of lake reclamation activities and belonged to three main categories: illegal dykes constructed by fishermen for fishing, dykes constructed for the centralized elimination of *Oncomelania hupensis* snails (intermediate hosts of *Schistosoma japonicum*; Li et al., 2000), and ecological dykes constructed for the protection of waterbirds (Zou et al., 2019). Lake reclamation has produced a series of serious and far‐reaching environmental impacts, such as reduced hydrological connectivity (Nakayama & Watanabe, 2008), sedimentation (Xu et al., 2017), and biodiversity reduction (Fang et al., 2006). Therefore, adhering to the concept of sustainable development, the Chinese government proposed “to step up conservation of the Yangtze River and stop its overdevelopment” for the Yangtze River Economic Belt (Xiang et al., 2021). To repair the ecological damage of Dongting Lake caused by lake reclamation, the Hunan Provincial Government completely demolished 459 illegal dykes before the end of 2017 and preserved 14 ecological dykes for biodiversity protection and flood control. However, there has been no systematic evaluation of whether the massive demolition of the dykes has improved the ecological services of the wetlands or ultimately led to a significant increase in biodiversity, as expected by government agencies.

The impacts of dykes on wetland ecosystems are primarily related to habitat management, with dyked wetlands providing sufficient food resources to maintain high waterbird diversity by regulating water levels (Murkin et al., 1982; Wang et al., 2020). Although the dykes in Dongting Lake were demolished on the basis of “dykes have negative effects on waterbird diversity”, studies have demonstrated that “ecological dykes have positive effects on maintaining waterbird diversity” (Zhang, Zhang, et al., 2021), which seems to be contradictory. Certainly, the government has preserved 14 ecological dykes, which fully illustrates the positive effect of ecological dykes. However, of the 459 dykes in Dongting Lake that were abruptly demolished, a careful pre‐assessment of which dykes could be offering important ecosystem services is lacking. Therefore, there is an urgent need to comprehensively evaluate the effects of dyke demolition, especially for those dykes that have proven to be ecologically important for waterbird diversity.

Biodiversity remains relatively stable under slow changes in environmental drivers (Scheffer & Carpenter, 2003). However, stability is disrupted under sudden drastic disturbance (Scheffer et al., 2001); for example, under the dyke demolition in our study area. As environmental drivers approach the critical point of ecological thresholds, communities change abruptly, with a series of species becoming extinct immediately and some gradually disappearing (Bestelmeyer et al., 2011; Dakos & Bascompte, 2014). Several aspects of waterbird communities vary with space and time, especially when subjected to external environmental pressures; thus, a large number of diversity indicators are needed to quantify these variables. Species richness and number, species proportions, and foraging guild classification are commonly used metrics for analyzing the diversity of waterbirds in different habitats (Fan et al., 2021; Zhang et al., 2018; Zhang, Zhang, et al., 2021; Zou et al., 2019). Richness alone may obscure the internal dynamics of species composition, but species turnover provides further insight into the rate at which species gains and losses over time (Poysa et al., 2019). All these indices are useful measures to quantify the response of waterbird communities to habitat destruction. Therefore, it is highly desirable to evaluate the response of waterbirds to abrupt habitat changes based on indicators such as species richness, abundance, species composition, and species turnover.

Currently, changes in waterbird diversity due to dyke demolition in the Dongting Lake wetlands have not been comprehensively assessed. Using the East Dongting Lake wetland as an example, this study aims to: (1) reveal the effects of dyke demolition on habitat factors by comparing the differences in habitat factors in dyke‐demolished and dyke‐preserved areas; (2) assess changes in waterbird diversity due to dyke demolition based on pre‐ and post‐demolition monitoring data; and (3) explain the key mechanisms of dyke demolition impact on the waterbird diversity by identifying crucial factors for waterbirds’ differential use of dyke‐demolished and preserved areas. The results of this study provide insights for future wetland restoration in Dongting Lake, as well as suggestions for priority areas for waterbird diversity conservation.

## 2 MATERIALS AND METHODS

### 2.1 Study area

**FIGURE 1:**
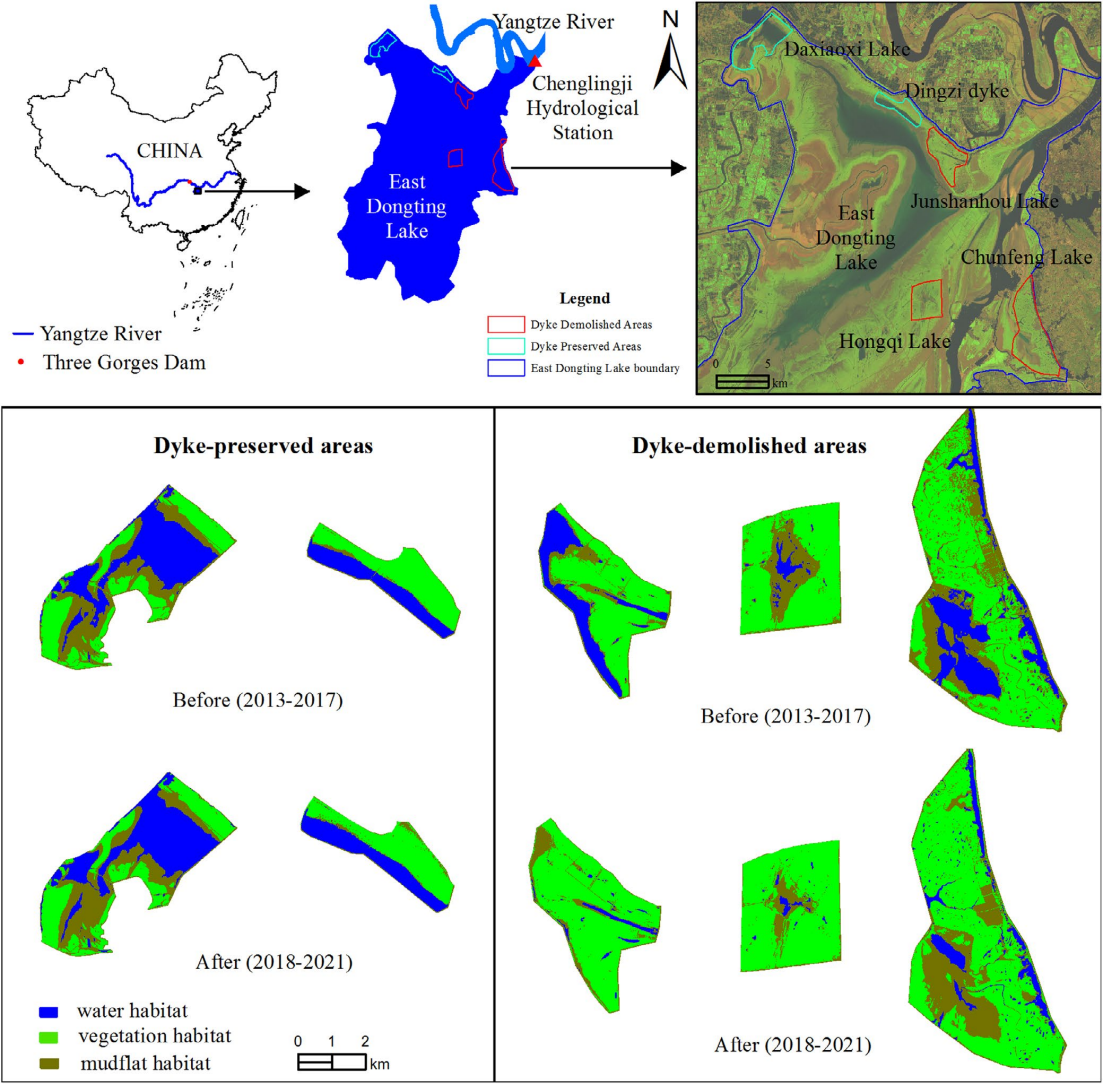
Study area and habitat changes before and after dyke demolition

Dongting Lake is the key Yangtze River floodplain wintering region for migratory waterbirds along the East Asian–Australasian Flyway (Cao et al., 2008). Large seasonal water level fluctuations (from 36 to 20 m) and abnormal (in particular, too early) water recession has threatened these habitats (Guan et al., 2016; Zhang et al., 2020). Five sub‐lakes (Dingzi Dyke and Daxiaoxi, Chunfeng, Hongqi, and Junshanhou lakes), all of which are crucial wintering sites for waterbirds in the Dongting Lake wetlands (Zou et al., 2019), were selected for study (Figure 1). Dykes distributed around each lake (with elevations of approximately 26 m) could mitigate the effects of hydrological change on habitats, providing diverse and stable habitats for waterbirds (Zhang, Zhang, et al., 2021). However, the dykes in Chunfeng, Hongqi, and Junshanhou lakes were all demolished, as reported by the sixth group of the Central Environmental Protection Inspectorate and directed by the Hunan Provincial Government in 2017. As such, Chunfeng, Hongqi, and Junshanhou lakes were classified as dyke‐demolished areas, while Daxiaoxi Lake and Dingzi Dyke were classified as dyke‐preserved areas.

The five study areas comprise approximately 6300 ha (4400 ha for dyke‐demolished areas and 1900 ha for dyke‐preserved areas) and include three major habitats—vegetation, mudflat, and water habitats (including shallow water with water depths of <20 cm)—which transition from high to low elevations in each area. These habitats provide important feeding grounds for waterbirds (e.g., water for fish eaters and omnivores, shallow water for insectivores, and vegetation for herbivores and tuber feeders; Zhang et al., 2021; Zou et al., 2019), but were significantly changed by dyke demolition (Figure 1; details presented in the Result section).

### 2.2 Bird survey and analysis

Waterbird survey data from the 2012/2013 to 2020/2021 wintering periods were collected. Under fine weather conditions in mid‐January (coinciding with the population peak of wintering waterbirds in the Dongting Lake wetland), with the assistance of the Administrative Bureau of East Dongting Lake Nature Reserve, several groups of investigators conducted simultaneous waterbird surveys in the five study areas (Figure 1) according to the same protocol. Waterbirds were recorded using the absolute number counting method (Cimprich, 2009). All observed wintering waterbird species are listed in Appendix S1.

To assess the influence of dyke demolition as a function of species, species were aggregated into five foraging guilds—tuber feeders, omnivores, insectivores, herbivores, and fish eaters—based on their distinct feeding habits (Zhang et al., 2016; Zou et al., 2019). According to historical data and previous studies (Cao et al., 2008), the East Dongting Lake wetlands serve as the major wintering habitats of waterbirds, with dominant species including Bean goose *Anser fabalis* and Falcated Duck *Mareca falcata*; the site also supports threatened species (listed as critically endangered, endangered, and vulnerable on the International Union for Conservation of Nature (IUCN) Red List (IUCN, 2021). Tracking the population changes of dominant or threatened species and their relationship with the habitat use can further determine the response of waterbirds to habitat changes. Six dominant and threatened species, whose population (the percentage of individuals) was significantly changed after dyke demolition, were selected to determine the correlation between waterbirds and their habitat use at the species level (Appendix S1).

Richness (number of species) and abundance (waterbird individuals) are widely used to evaluate the waterbird population change; however, abundance might be more sensitive to habitat change (Ma et al., 2009). For migratory birds, annual variation of the raw value of abundance may not truly reflect the influence of habitat change on waterbird populations in wintering sites. For example, breeding success and food availability at stopover sites may also impact the annual variation (Baker et al., 2004; Tidwell et al., 2013). Given the significant annual fluctuations in wintering waterbird abundance in Dongting Lake, the percentage of waterbird individuals, rather than abundance, might be a better proxy for evaluating the waterbird responses to dyke demolition (Zhang et al., 2020). Therefore, in this study, the number of species and the percentage of waterbird individuals were both used to evaluate the waterbird response to dyke demolition. The percentages of waterbird individuals in the dyke‐demolished/preserved areas were calculated by the number of individuals in the dyke‐demolished/preserved areas divided by the total individuals across the whole study area.

### 2.3 Habitat variables

Habitat variables, including water area, shallow water area, mudflat area, vegetation area (sedge [*Carex* spp.] meadow area), and the normalized difference vegetation index (NDVI) of the vegetation were extracted from satellite images from 2013 to 2021 (Appendix S2). A total of 68 Landsat 5/8 or Sentinel‐2 satellite images obtained from 2013 to 2021 were used to evaluate the changes in the areas of three habitats along water level gradients in both dyke‐preserved areas and dyke‐demolished areas before (2013–2017) and after (2018–2021) dyke demolition. Images acquired from mid‐winter (January–February, with a similar period of waterbird surveys) from 2013 to 2021 were used to evaluate the impact of habitat changes on waterbird populations (Appendix S2). According to Zou et al. (2019), different habitat (vegetation, mudflat, and water) areas were extracted by the decision tree classification method with the help of the NDVI and the modified normalized difference water index (MNDWI), followed by classification accuracy evaluation for each image using a standard error matrix (confusion matrix; Dadaser‐Celik et al., 2008). After accuracy assessment of the individual classifications, the overall accuracies of all classifications (2011–2021, using reference data from the images) were >93%, while the kappa statistic values for the same classifications were >0.91. Limited by insufficient Landsat 5/8 images in the early overwintering period (November) and mid‐overwintering period (January, similar to the period of waterbird surveys), the NDVI of vegetation that represents the food availability of geese was calculated from the MOD09Q1 dataset at a spatial resolution of 250 m and 8‐day intervals (Terra MODIS images provided by the Earth Resources Observation Systems [EROS] data center, the United States Geological Survey [USGS]).

Specific feeding habitat requirements are driven by bird morphology; for example, owing to the lengths of the tarsometatarsi or necks (Collazo et al., 2002; Darnell & Smith, 2004; Ntiamoa‐Baidu et al., 1998; Poysa, 1983), insectivores guilds and shorebirds (e.g., Spotted redshank, Dunlin, and Northern lapwing) prefer shallow water habitats (water depths of <20 cm; Zhang, Zhang, et al., 2021; Zou et al., 2019). Therefore, according to the methods presented in Zou et al. (2019) and Zhang, Zhang, et al. (2021), shallow water habitats in the dyke‐demolished and preserved areas were extracted and calculated as the water level minus the elevation at each point.

### 2.4 Statistical analysis

To further quantify the temporal variability of species composition in dyke‐demolished and preserved areas, we calculated and plotted turnover over time. The calculation of turnover is based on the original formula of Macarthur and Wilson (1963), which was modified by Diamond (1969) to express proportional turnover to compare changes in bird richness over different years on the same island. The R package (codyn) developed by (Hallett et al., 2016) calculates the species turnover between two time periods; that is, the proportion of species gains and losses observed at both time points. We calculated the proportions of species gains and losses in the dyke‐demolished and preserved areas from 2013 to 2021.

$$\text{Total turnover}=\frac{\text{Species gains}+\text{Species losses}}{\text{Total species observed at two ‐ time points}}$$

Species turnover was performed using presence–absence data, and all statistical calculations were performed using the R package codyn (Hallett et al., 2016).

All data were assessed for normality using the Kolmogorov–Smirnov test. An independent sample *t*‐test was used for normally distributed data, whereas the Mann–Whitney U test was used for non‐normally distributed data. In all the statistical results, statistical significance was set at *p* < .05. The Kolmogorov–Smirnov test, independent sample *t*–test, and Mann–Whitney U test were performed using IBM SPSS Statistics version 23.0.

## 3 RESULTS

### 3.1 Habitat change before and after dyke demolition

**FIGURE 2:**
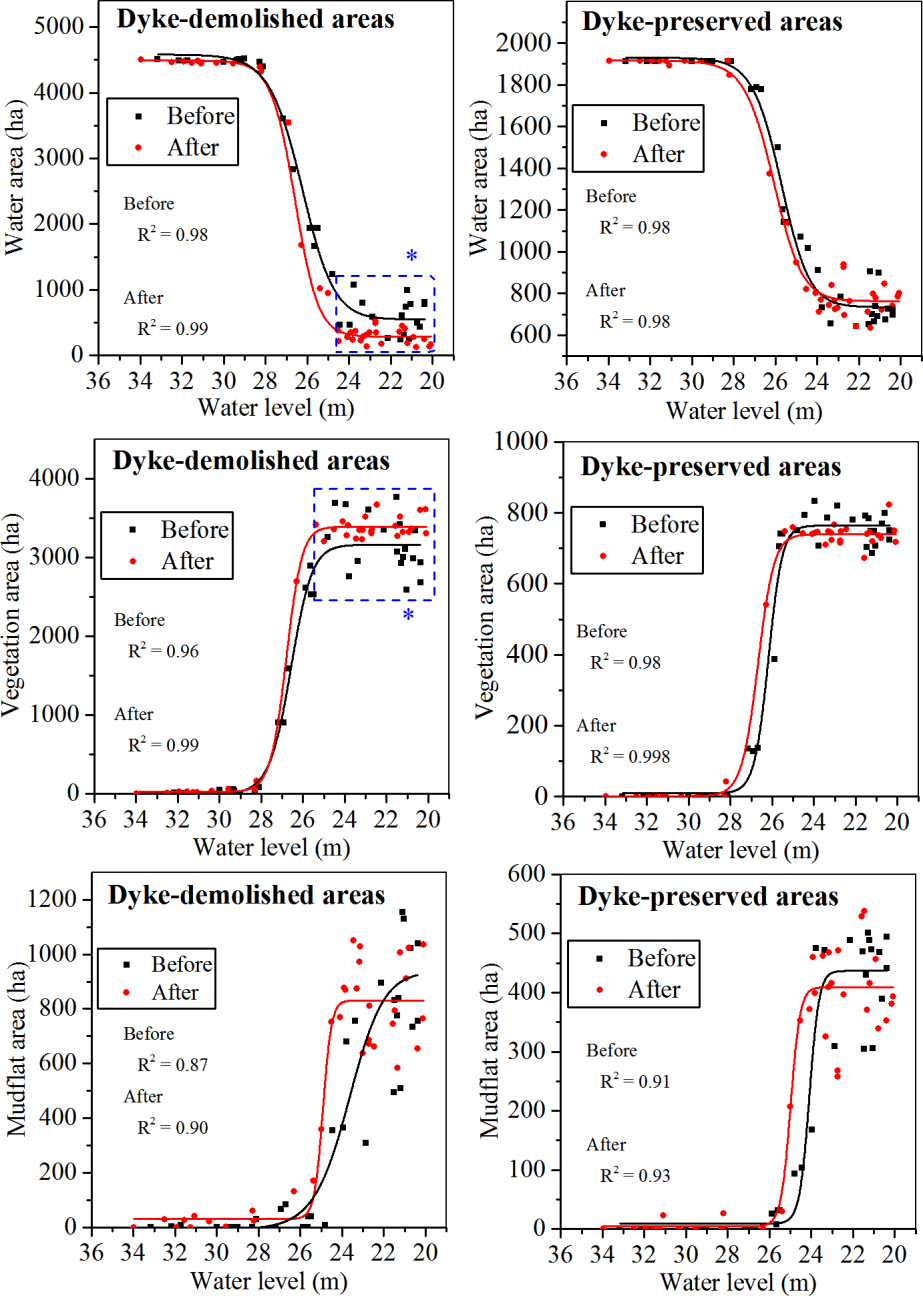
Changes in the areas of three habitats along water level gradients in the dyke‐preserved and dyke‐demolished areas before (2013–2017) and after (2018–2021) dyke demolition. The black dots and lines represent the average habitat area from 2013 to 2017 (before dyke demolition), and the red dots and lines represent the average habitat area from 2018 to 2021 (after dyke demolition). The water level is the current day's Chenglingji water level corresponding to the satellite image. * denotes *p* < .05

In the dyke‐demolished areas, water levels dropped to <28 m and water, mudflat, and vegetation habitats showed obvious changes before and after dyke demolition (Figure 2). Specifically, as the water level declined, the water area declined; when the water level was <25 m, the water area in dyke‐demolished areas was significantly lower than that before the demolition (*p* < .05; Figure 2). The vegetation and mudflat habitats showed increasing trends, but when the water level was <25 m, only vegetation in the dyke‐demolished areas was significantly higher than that before the demolition (vegetation area: *p* < .05; mudflat area: *p* > .05; Figure 2). Comparatively, in the dyke‐preserved areas, the areas of water, mudflat, and vegetation habitats remained stable as the water level remained relatively stable (Figure 2). In summary, dykes can delay water recession and vegetation exposure, and play a “water storage” role. After dyke demolition, water receded faster, resulting in the early exposure and growth of vegetation, and the significant increase in vegetation area.

**FIGURE 3:**
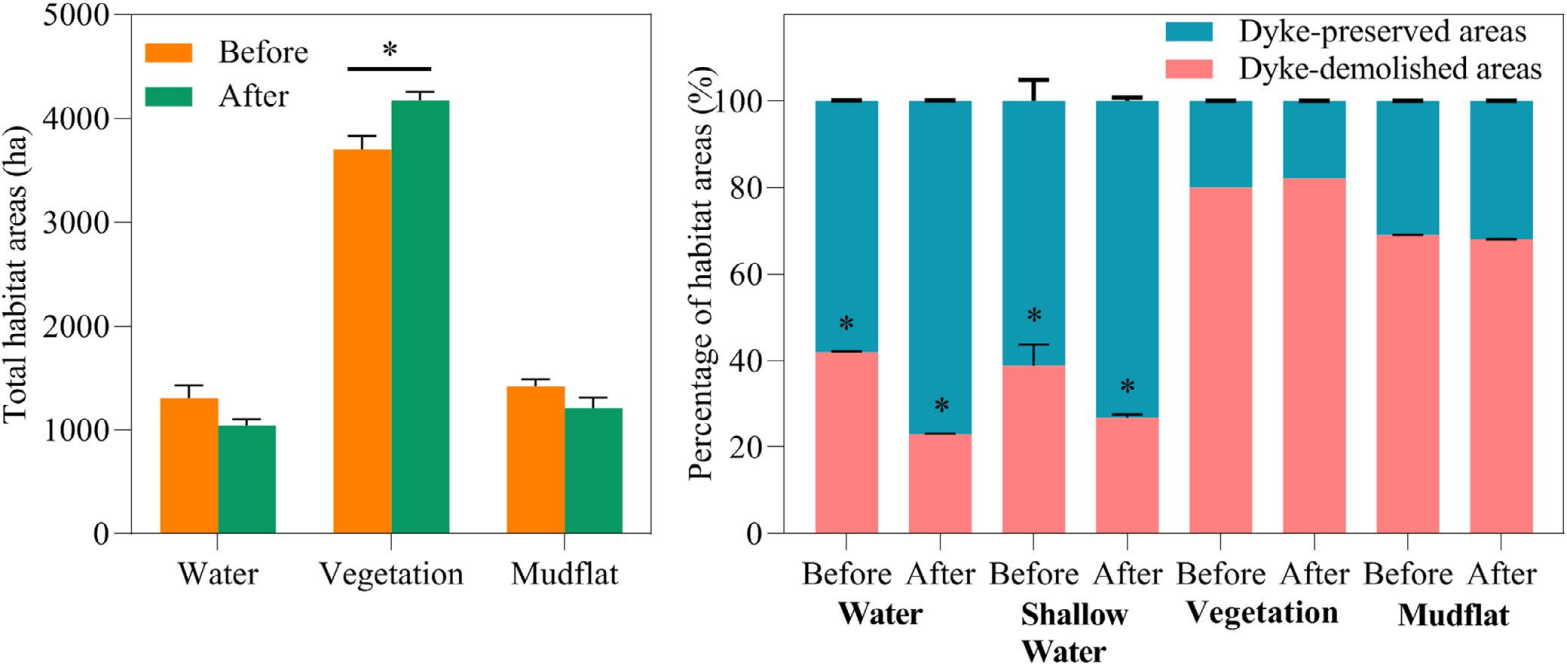
Comparison of changes in total habitat areas and percentages of habitat areas before and after dyke demolition. Error bars indicate the standard error of the mean. * denotes *p* < .05

Dyke demolition changed the total areas of habitats in the study areas. Vegetation significantly increased (*t*‐test, *t* = 2.794, *p* < .05; Figure 3); and water and mudflat exhibited declining trends that were not statistically significant (both *p* > .05; Figure 3). The percentage areas of water and shallow water both significantly decreased in the demolished areas after dyke demolition (*t*‐test, water, *t* = −2.794, *p* < .05; shallow water, *t* = −2.46, *p* < .05; Figure 3), but significantly increased in the dyke‐preserved areas (*t*‐test, water, *t* = 2.794, *p* < .05; shallow water, *t* = 2.46, *p* < .05; Figure 3). The percentage area of vegetation significantly increased in the demolished areas after dyke demolition (*t*‐test, *t* = −4.051, *p* < .05; Figure 3), but significantly decreased in the dyke‐preserved areas (*t*‐test, *t* = 4.051, *p* < .05; Figure 3). Significant changes were not observed in the percentage areas of mudflat in the dyke‐demolished and preserved areas after dyke demolition (all *p* > .05, Figure 3). Notably, in the dyke‐demolished areas, the percentage areas of water and shallow water both decreased by >30% after dyke demolition (water, from 42% to 23%; shallow water, from 38.85% to 26.80%).

### 3.2 Changes in species composition of waterbird communities

**FIGURE 4:**
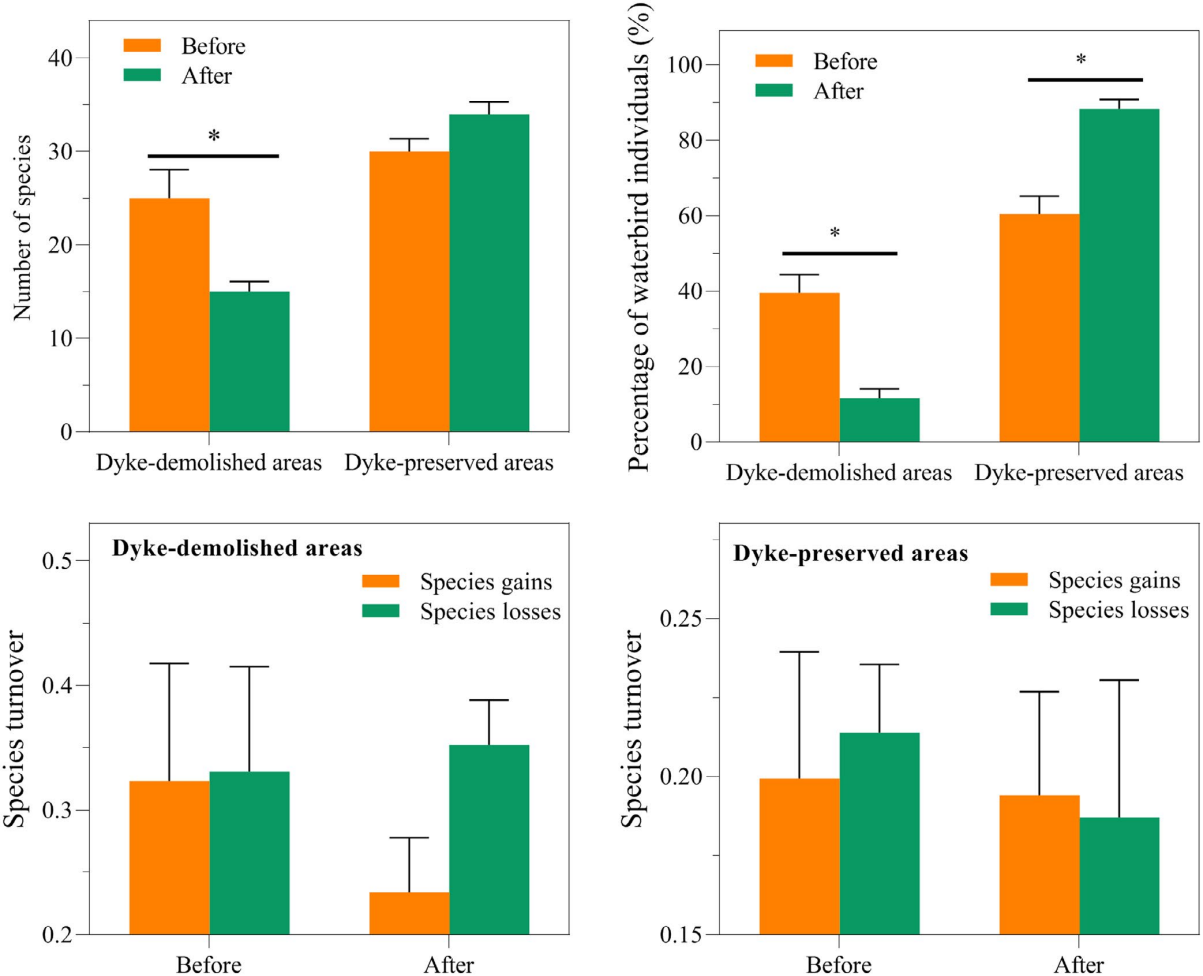
Changes in the number of species, percentage of waterbird individuals, and species turnover in dyke‐demolished and preserved areas before and after dyke demolition. * denotes *p* < .05

At the community level, a total of 452,021 individuals, corresponding to 62 species, were recorded in the entire study area during the winter seasons of 2012/2013–2020/2021 (Appendix S1). Among these, 50 species (126,100 individuals) were recorded in the dyke‐demolished areas, including nine rare species (Appendix S1), while 55 species (325921 individuals) were recorded in dyke‐preserved areas, including 14 rare species (Appendix S1). In the dyke‐demolished areas, both the number of species and the percentage of waterbird individuals significantly decreased after dyke demolition (number of species: decreased by 40%, *t* = 3.046, *p* < .05; percentage of waterbird individuals: decreased by 70%, *t* = 4.776, *p* < .05; Figure 4). In the dyke‐preserved areas, no significant difference was observed in the number of species between the pre‐ and post‐demolition periods (*t* = −1.683, *p* > .05; Figure 4), while the percentage of waterbird individuals significantly increased in the post‐demolition period (*t* = −4.776, *p* < .05; Figure 4). Changes in waterbird community composition might be caused by the changes in the relative values of “species gains” and “species losses” (Figure 4). After dyke demolition, the “species losses” tended to be higher than the “species gains” in dyke‐demolished areas, although this did not reach the statistically significant level of 0.05 (Figure 4). Interestingly, compared with waterbird richness (as represented by the number of species), waterbird abundance (as represented by the percentage of waterbird individuals) was more sensitive to dyke demolition (Figure 4).

**FIGURE 5:**
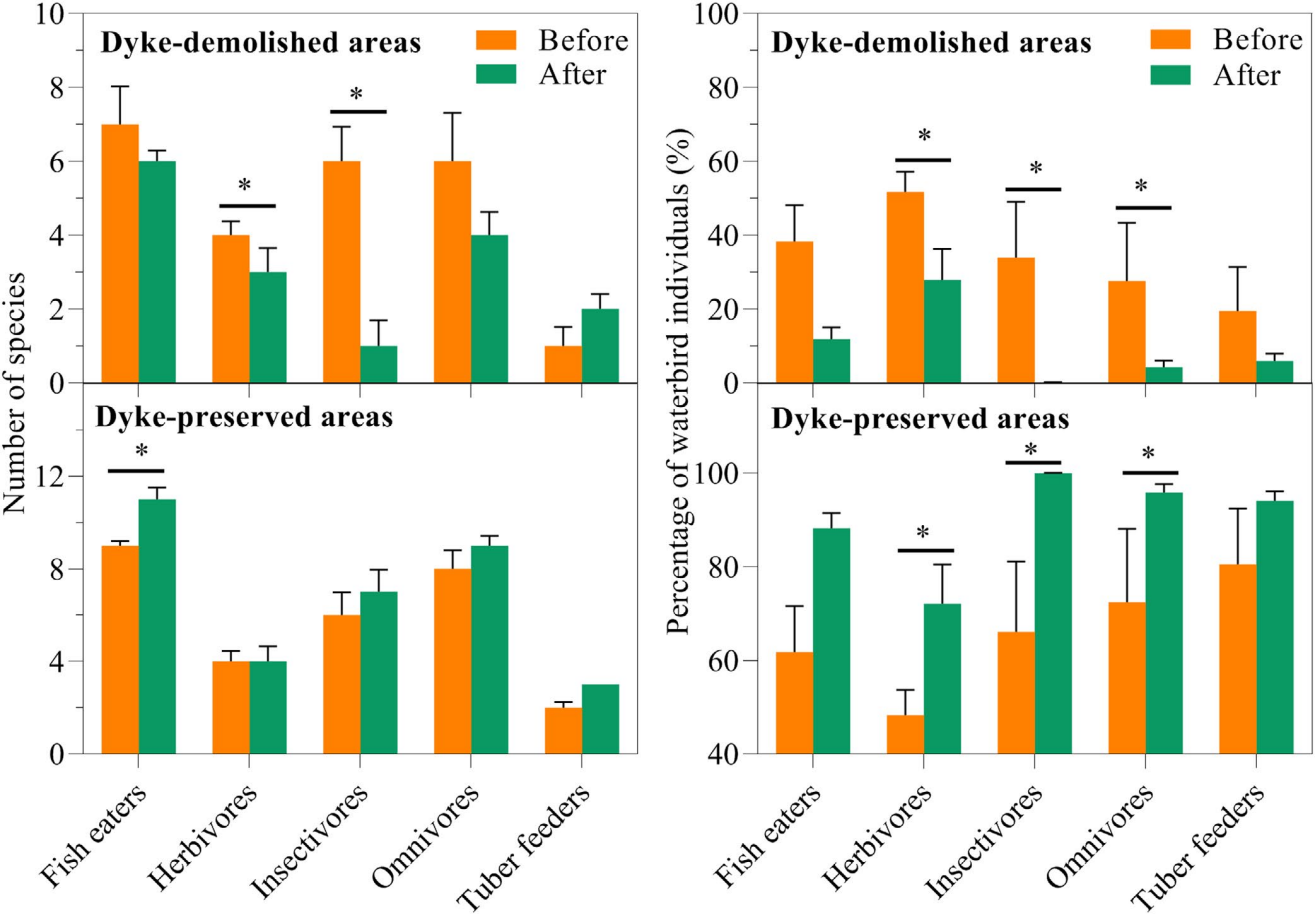
Changes in the number of species and percentage of individuals of five foraging guilds in dyke‐demolished and preserved areas before and after dyke demolition. * denotes *p* < .05

At the foraging guild level, both the number of species and the percentage of individuals of the five foraging guilds showed similar trends as at the community level (Figures 4 and 5); that is, declining trends in dyke‐demolished areas but increasing trends in dyke‐preserved areas (Figure 5). Specifically, the number of species of herbivores and insectivores significantly decreased in the dyke‐demolished areas (*t*‐test, herbivores: *t* = 2.401, *p* < .05; insectivores: *t* = 4.422, *p* < .05; Figure 5). In the dyke‐preserved areas, the number of species of fish eaters significantly increased in the post‐demolition period (Mann–Whitney U test, fish‐eaters: u = 2, *p* < .05; Figure 5). The percentage of waterbird individuals of herbivores, insectivores and omnivores significantly decreased in the dyke‐demolished areas (Mann–Whitney U test, insectivores: u = 0, *p* < .05; omnivores: u = 2, *p* < .05; herbivores: *t*‐test, *t* = 2.495, *p* < .05; Figure 5), but significantly increased in the dyke‐preserved areas (Mann–Whitney U test, insectivores: u = 0, *p* < .05; omnivores: u = 2, *p* < .05; herbivores: *t*‐test, *t* = −2.494, *p* < .05; Figure 5).

**FIGURE 6:**
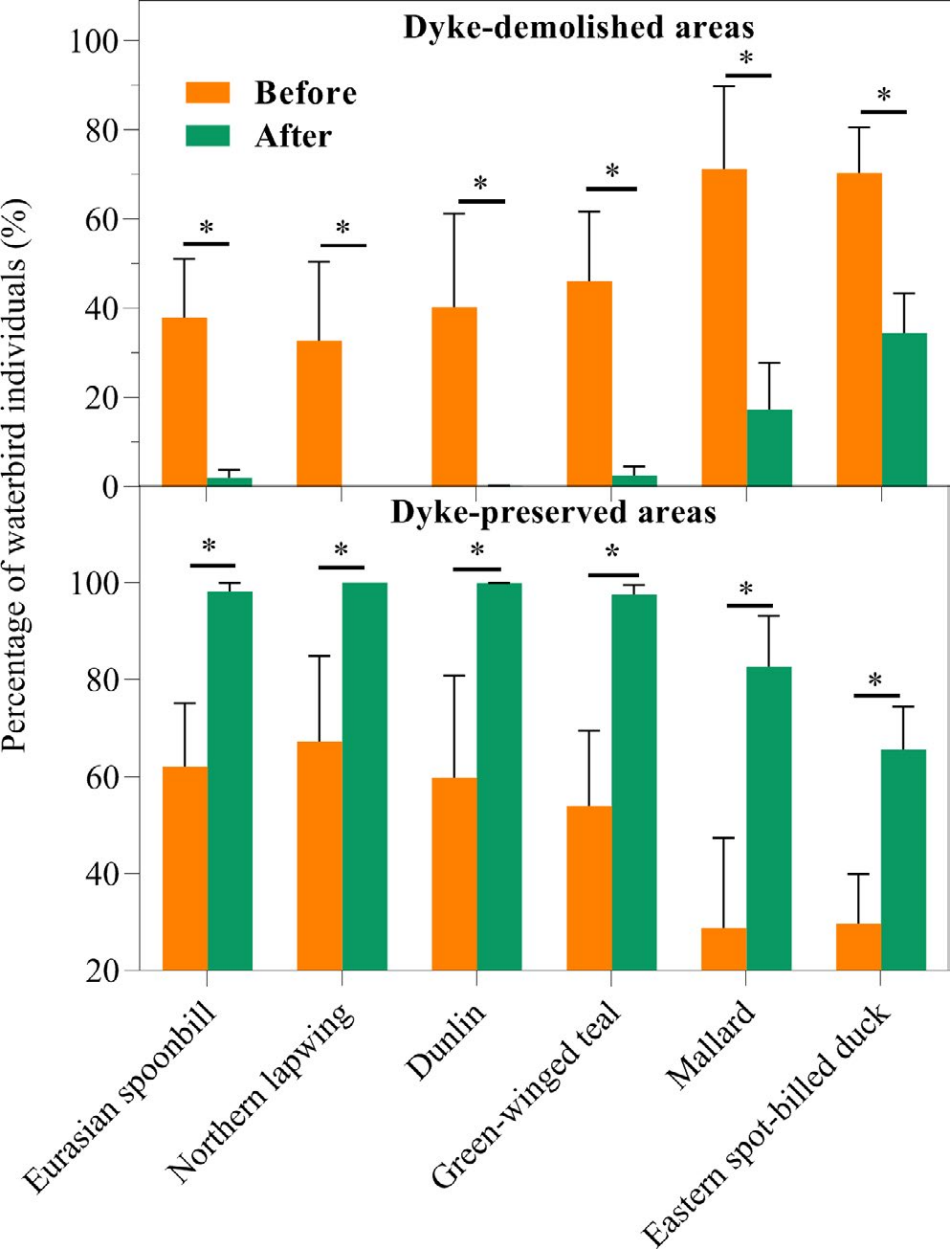
Changes in the percentage of individuals of six species in dyke‐demolished and preserved areas before and after dyke demolition. * denotes *p* < .05

At the species level, populations from six species (as represented by the percentage of individuals) exhibited significant declining trends in the dyke‐demolished areas but significant increasing trends in the dyke‐preserved areas (Figure 6). Specifically, the percentages of individuals of the six species in the dyke‐demolished areas significantly decreased after dyke demolition (Figure 6), including Dunlin (Mann–Whitney U test, u = 0, *p* < .05), Eurasian spoonbill (*t*‐test, *t* = 2.728, *p* < .05), Northern lapwing (Mann–Whitney U test, u = 2, *p* < .05), Mallard (*t*‐test, *t* = 2.346, *p* < .05), Green‐winged teal (*t*‐test, *t* = 2.769, *p* < .05) and Eastern spot‐billed duck (*t*‐test, *t* = 2.560, *p* < .05). Moreover, four species—Eurasian spoonbill, Northern lapwing, Dunlin, and Green‐winged teal—nearly disappeared (percentage of individuals <2.5%) from dyke‐demolished areas after dyke demolition (Figure 6). In contrast, the percentages of individuals of the six species significantly increased in the dyke‐preserved areas in the post‐demolition period (*t*‐test, all *p* < .05; Figure 6).

### 3.3 Habitat variables causing differences in waterbird population distribution

**FIGURE 7:**
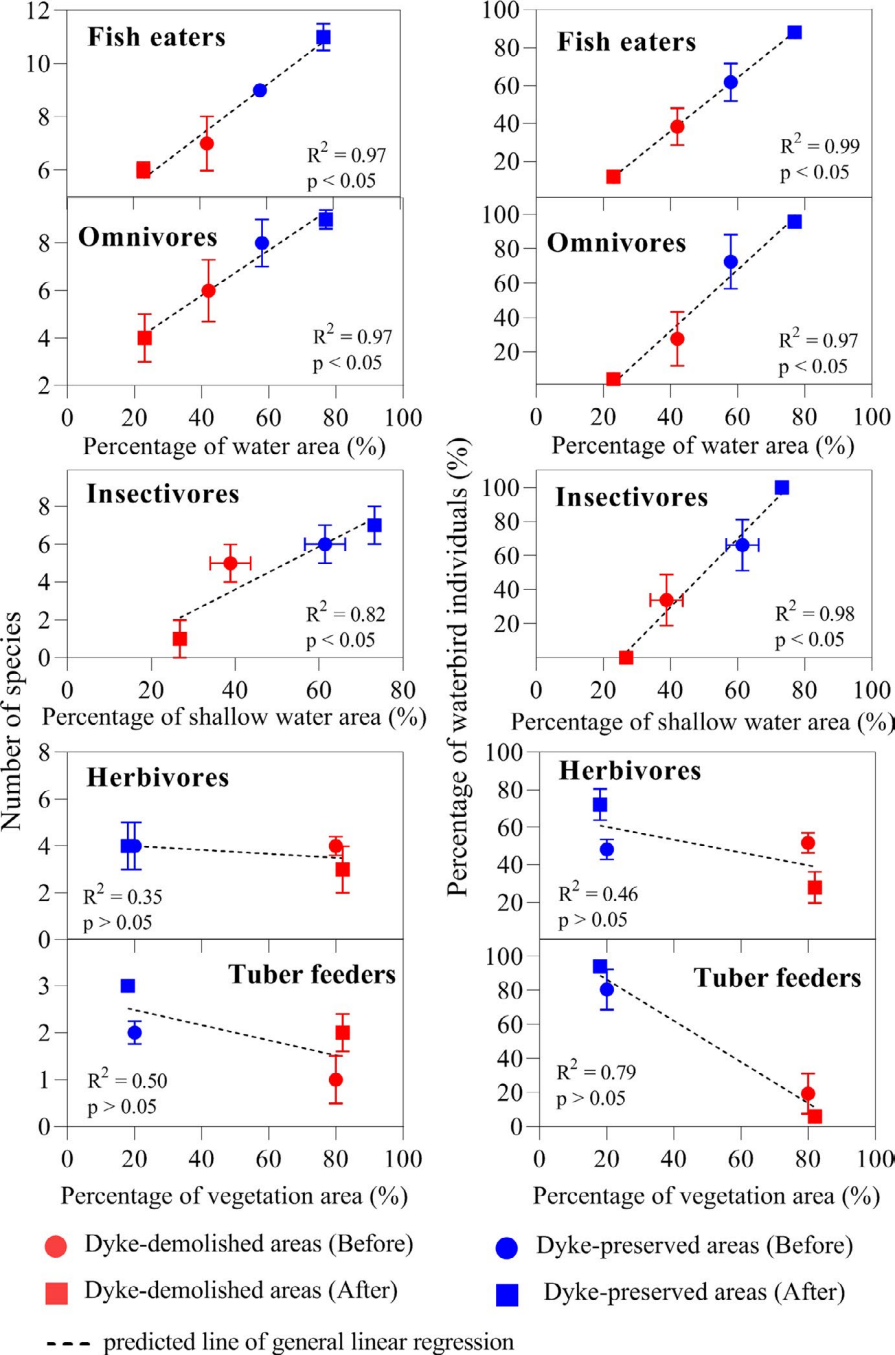
Relationships between the number of species and the percentage of waterbird individuals of five foraging guilds and their suitable habitat variables. Error bars represent the standard error (SE)

At the foraging guild level, the changes in the percentage of water area were crucial habitat factors for water‐dependent foraging guilds (including fish eaters and omnivores) pre‐ and post‐demolition as indicated by the significant positive correlations between the number of species and percentage of waterbird individuals of such guilds and their habitat areas (the number of species, fish eaters: *R* ^2^ = 0.97, *p* < .05; omnivores: *R* ^2^ = 0.97, *p* < .05; percentage of waterbird individuals, fish eaters: *R* ^2^ = 0.99, *p* < .05; omnivores: *R* ^2^ = 0.97, *p* < .05; Figure 7); that is, the number of species and the percentages of fish eaters, and omnivores all decreased with the sharp decline in the percentage of water areas in dyke‐demolished areas, but increased with the drastic increase in the percentage of water areas in the dyke‐preserved areas (*t*‐test or Mann–Whitney U test, all *p* < .05; Figure 7). The changes in the percentage of shallow water area were crucial habitat factors for insectivores guild pre‐ and post‐demolition as indicated by the significant positive correlations between the number of species and percentages of waterbird individuals of such guild and its habitat area (insectivores, the number of species: *R* ^2^ = 0.82, *p* < .05; percentage of waterbird individuals: *R* ^2^ = 0.98, *p* < .05; Figure 7). Compared with the number of species, the percentages of waterbird individuals of water‐ and shallow water‐dependent foraging guilds were more sensitive to the changes in water areas (including shallow water; Figure 7). Although the percentages of vegetation areas increased in the dyke‐demolished areas and decreased in the dyke‐preserved areas, positive correlations were not observed in the species number and percentages of waterbird individuals of the vegetation‐dependent foraging guilds (including herbivores and tuber feeders) after dyke demolition (negative correlations, all *p* > .05; Figure 7). In summary, except for herbivores and tuber feeders, changes in the number of species and percentages of individuals of fish eaters, insectivores, and omnivores probably reflect the drastic changes in the percentage of water habitats (including shallow water) after dyke demolition.

**FIGURE 8:**
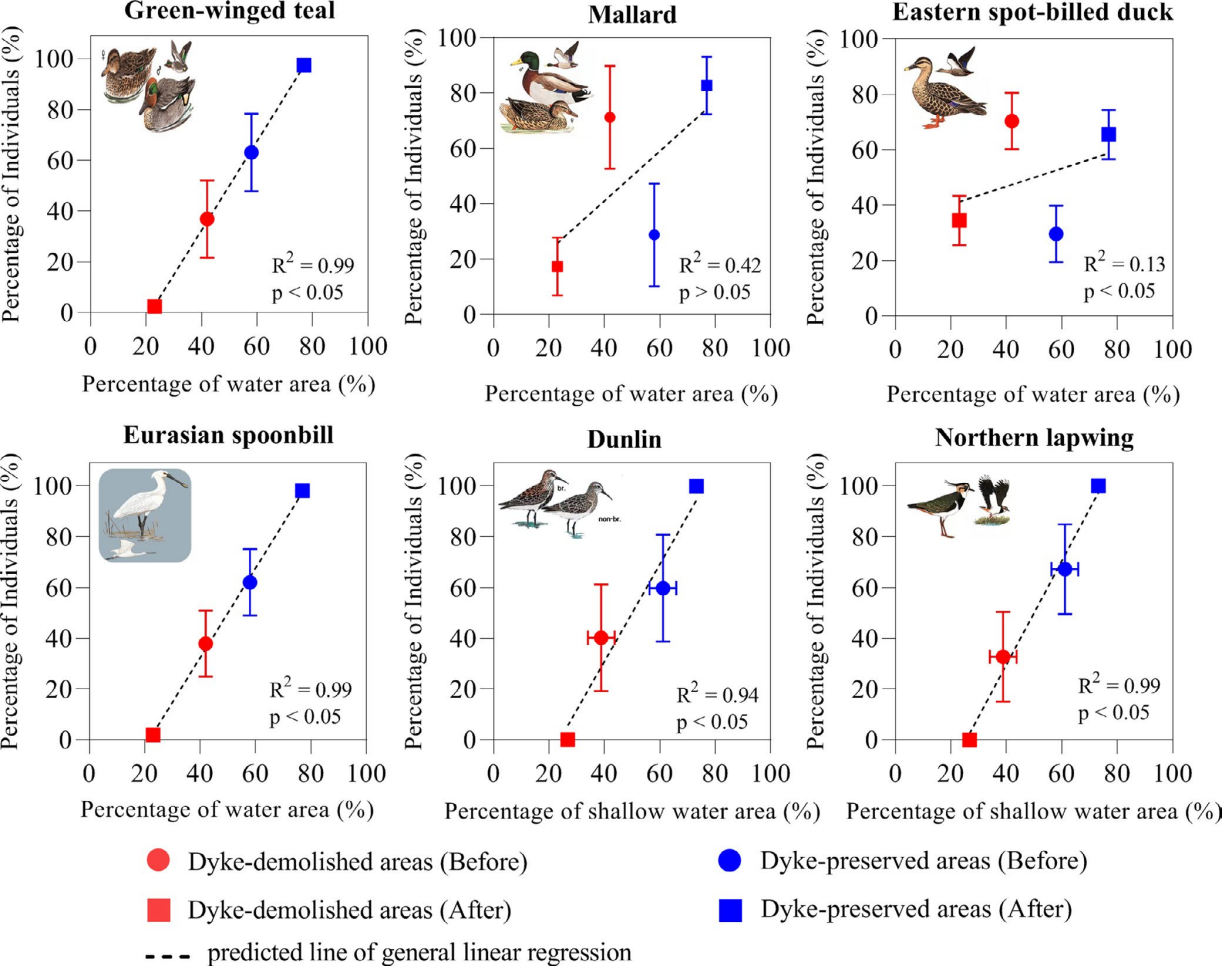
Relationships between the percentages of individuals of six waterbird species with significant variation and their suitable habitat variables. Error bars represent the standard error (SE)

At the species level, among the six selected species, percentages of four species were positively correlated with changes in the percentage areas of their corresponding suitable habitats (Green‐winged teal: *R* ^2^ = 0.99, *p* < .05; Eurasian spoonbill: *R* ^2^ = 0.99, *p* < .05; Dunlin: *R* ^2^ = 0.94, *p* < .05; Northern lapwing: *R* ^2^ = 0.99, *p* < .05; Figure 8). Specifically, the percentages of two water‐dependent species (Green‐winged teal and Eurasian spoonbill) all decreased along with the sharp decline in the percentage of water area in the dyke‐demolished areas, but increased along with the drastic increase in the percentage of water area in the dyke‐preserved areas (*t*‐test or Mann–Whitney U test, all *p* < .05; Figure 8). The percentages of two shallow water‐dependent species (Dunlin and Northern lapwing) decreased along with the sharp decline in the percentage of shallow water in the dyke‐demolished areas, but increased along with the drastic increase in the percentage of shallow water in the dyke‐preserved areas (*t*‐test or Mann–Whitney U test, all *p* < .05; Figure 8). Significant positive correlations were not observed between changes in percentages of Mallard and Eastern spot‐billed duck and the percentage of water areas after dyke demolition (Mallard: *R* ^2^ = 0.42, *p* > .05; Eastern spot‐billed duck: *R* ^2^ = 0.13, *p* > .05; Figure 8). In summary, except for Mallard and Eastern spot‐billed duck, changes in the percentages of water‐dependent species (including shallow water‐dependent species) probably reflect the drastic changes in the percentage of water habitats after dyke demolition.

## 4 DISCUSSION

Based on long‐term monitoring data of waterbirds, by employing the number of species and the percentage of waterbird individuals, this study investigated the spatiotemporal distribution dynamics of waterbirds due to changes in habitat hydrology and food availability following dyke demolition. We found that dykes can delay water recession; however, the water receded faster after dyke demolition, resulting in drastic changes in habitat areas, and in particular a sharp decline in the percentage area of water and shallow water habitats in dyke‐demolished areas (water from 42 to 23%; shallow water from 38.85 to 26.80%; Figure 3). In contrast, the dyke‐preserved areas maintained relatively stable area percentages for the three habitat types (Figure 3). We also observed strong differences in the spatiotemporal distribution dynamics of waterbirds, which may be due to the sudden disruption of the relatively stable balance of “hydrology–habitat–waterbird diversity” caused by the demolition of dykes in East Dongting Lake. Given the distinct feeding requirements of specific waterbird assemblages (Bellio et al., 2009), different habitat conditions caused by dyke demolition (Figure 3) likely led to different waterbird compositions and distributions (Figures 5 and 6), as indicated by the relationships between waterbird populations and habitat changes (Figures 7 and 8).

Changes in the water habitats after dyke demolition probably led to changes in the distributions of the water‐dependent foraging guilds, including fish eaters, insectivores, and omnivores. Suitable habitats for these guilds were generally water habitats, while water depth requirements generally varied according to feeding habits (Bellio et al., 2009; Ma et al., 2010). For example, insectivores use shallow water as foraging habitats (Safran et al., 1997), while omnivores and fish eaters use deep water as foraging habitats (Afdhal et al., 2013; Paszkowski & Tonn, 2006). Previous studies showed that dyked wetlands can maintain larger and deeper water areas than un‐dyked wetlands (Monfils et al., 2014), providing suitable habitats for waterbirds that feed at different water depths, supporting great numbers of waterbirds (Weber & Haig, 1996; Zhang, Zhang, et al., 2021). In this study, we found that after the dykes were demolished, significant decreases occurred in the areas of the water habitats, including the shallow water habitats (Figure 2), resulting in a sharp decline in the number of species and percentages of water‐dependent guilds (including fish eaters and omnivores, such as Green‐winged teal, and Eurasian spoonbill; Figure 7 and Figure 8) and shallow water‐dependent guilds (insectivores, such as Dunlin, and Northern lapwing; Figure 7 and Figure 8). Our results show that dyke demolition can reduce the “water storage” function during the non‐flooding period (wintering period for migratory birds), leading to a sharp decline in water area and eventually reducing the populations of waterbirds that depend on this habitat; these waterbirds are forced into dyke‐preserved areas that provide sufficient suitable habitats.

Changes in vegetation areas after dyke demolition are unlikely to be the reason for the drastic changes in the populations of herbivore guilds (Figure 7 and Figure 8), as inferred by the significant increase in vegetation area (Figure 3) but sharp decline in herbivore guilds in dyke‐demolished areas (Figure 5). Vegetation, and in particular sedge Carex meadows, are crucial foraging habitats for herbivores geese (Cong et al., 2012; Wang et al., 2013; Zhao et al., 2010, 2015). Compared with the quantity (i.e., vegetation area), the quality of sedge Carex meadows is more important to herbivore geese in the Dongting Lake wetland (Guan et al., 2016; Zou et al., 2019). Unusually early water recession in Dongting Lake (as indicated by the water level when the vegetation habitats were exposure), caused by the Three Gorges Dam and climate change, could have caused earlier germination and growth of sedge Carex meadows, resulting in a sharp decline in vegetation quality. This ultimately influenced herbivorous geese abundance and distribution (Guan et al., 2016; Zhang, Zou, et al., 2021; Zou et al., 2017). *Carex* spp. vegetation is generally distributed within a similar elevation to the dykes in the study area, and thus the dykes had limited effects on improving the habitat quality by delaying the germination and growth of *Carex* spp., and ultimately failed to attract more geese (Zhang, Zhang, et al., 2021). Moreover, after dyke demolition, the day of year (DOY) when the NDVI of vegetation reached the maximum value was significantly earlier (before DOY 300) in dyke‐demolished areas (Appendix S3) than in dyke‐preserved areas (also before DOY 300, Appendix S3), but the difference was not significant. This suggests that when geese arrive at Dongting Lake (DOY 305), vegetation is already in the phenological withering period (Liang et al., 2021; Zhang, Zou, et al., 2021), which ultimately influences the distribution of herbivores (Figures 5 and 6). In summary, the quality of vegetation was both affected by dyke demolition and inter‐annual variation of hydrological regimes in Dongting Lake. Future research will focus on the reasons for the decrease in the percentages of herbivores (e.g., Bean geese) in the dyke‐demolished areas.

Compared with the significant changes in other foraging guilds before and after dyke demolition, relatively small and extremely few species of tuber feeder waterbirds changed (only common crane and tundra swan, Appendix S1). This was probably due to the relatively small number of species and the number of tuber feeder waterbirds in this study area (a total of 6 species with 5,634 individuals were observed; of these, 5 species with 659 individuals were accounted for in the dyke‐demolished areas) and were not the dominant species in the East Dongting Lake; however, we still analyzed the impact of dyke demolition on this guild owing to them being endangered. The dominant food items of the cranes include *Polygonum criopolitanum* and *Potentilla limprichtii*, which grow in the bottomlands, whose above‐ground biomass varies significantly along the water level gradient (Hou et al., 2021; Zhang et al., 2013). The dominant food items of the tundra swan are submerged vegetation such as *Potamogeton crispus* and *Vallisneria natans* (Cong et al., 2011; Fox et al., 2011). Therefore, we speculate that the accelerated water recession caused by the dyke demolition might have led to a longer growth time for the major food of tuber feeders (e.g., cranes) in the mudflats and bottomlands area. This may have resulted in better food quality and ultimately an increased number of tuber feeders in the dyke‐demolished areas (Figure 5). In addition, the decreased water area in the dyke‐demolished areas may have caused a more rapid die‐off of previously less distributed submerged vegetation, and consequently reduced the population of tuber feeders that fed on it (Figure 5). Our results are similar to those related to the main wintering site (Poyang Lake) of tuber feeders (e.g., cranes and tundra swan), where water level affects food availability and waterbird abundance (Chen et al., 2016; Cong et al., 2011; Hou, Liu, et al., 2020).

This study focused on the factors related to the food resources of waterbirds, but not on the effects of inter‐annual variation in hydrological regimes, climate change, and human disturbance in Dongting Lake. These factors have been demonstrated to influence the habitat selection and spatiotemporal distribution of waterbirds (Adam et al., 2015; Wang et al., 2019; Yuan et al., 2014), and will be considered in future work.

## 5 CONCLUSIONS

Habitat suitability and accessibility for waterbirds depend on water level fluctuations, especially in periodically flooded wetlands (Baschuk et al., 2012; Li et al., 2019). Our study demonstrated that dyke demolition resulted in a significant reduction in wetland water areas under the same water level conditions. Furthermore, the large water area maintained in the dyke‐preserved areas over a long period further indicates the importance of the function of the dyke in storing water at low water levels.

The goal of dyke demolition is to interconnect the Dongting Lake hydraulic regime and restore its natural properties. This study only analyzed the effects of dyke demolition on wintering waterbird diversity and habitat factors, but it is unclear whether these effects have a cascading effect on the entire wetland ecosystem. Our results show that the species and number of wintering waterbirds in dyke‐demolished areas (Chunfeng Lake, Junshanhou Lake, and Hongqi Lake) declined year by year after dyke demolition, while the dyke‐preserved areas (Daxiaoxi Lake and Dingzi Dyke) showed an increasing trend. Meanwhile, the high temporal variability in species turnover underscores the importance of long‐term studies for our understanding of waterbird dynamics. Understanding the effects of water level fluctuations and adaptive water level management on waterbirds and vegetation communities at Dongting Lake can be facilitated by long‐term studies.

## 6 MANAGEMENT IMPLICATIONS

To strengthen wetland conservation and effectively restore wetland habitat quality for improving waterbird diversity, based on the present results, we propose the following recommendations: (1) constructing water habitats with different water depths for fish eaters, insectivores, and omnivores in dyke‐demolished areas; (2) adaptively regulating water levels in dyke‐preserved areas to provide diverse suitable habitats for waterbirds; (3) planting submerged plants for tuber feeders (e.g., Tundra Swan); and (4) increasing the height of dykes to delay water recession, and thereby synchronizing the exposure of *Carex* spp. vegetation and the arrival of geese.

## CONFLICT OF INTEREST

The authors declare no conflict of interest.

## AUTHOR CONTRIBUTION

**Feng Zhu:** Conceptualization (equal); Methodology (equal); Visualization (equal); Writing – original draft (equal). **Ye‐ai Zou:** Conceptualization (equal); Supervision (equal); Writing – review & editing (equal). **Pingyang Zhang:** Conceptualization (equal); Visualization (equal). **Siqi Zhang:** Conceptualization (equal); Visualization (equal). **Xinsheng Chen:** Conceptualization (equal); Visualization (equal). **Feng Li:** Formal analysis (equal); Funding acquisition (equal). **Zhengmiao Deng:** Funding acquisition (equal); Methodology (equal). **Hong Zhang:** Data curation (equal); Investigation (equal). **Zhibing Yu:** Data curation (equal); Investigation (equal). **Xiaoyong Zhu:** Data curation (equal); Investigation (equal). **Yonghong Xie:** Funding acquisition (equal); Methodology (equal). **Dongsheng Zou:** Funding acquisition (equal); Methodology (equal).

## Supporting information

Supplementary Material

## Data Availability

Relevant data in this study will be available via Dryad: https://doi.org/10.5061/dryad.fn2z34tvm

## References

[ece38782-bib-0001] Adam, M. , Musilova, Z. , Musil, P. , Zouhar, J. , & Romportl, D. (2015). Long‐term changes in habitat selection of wintering waterbirds: High importance of cold weather refuge sites. Acta Ornithologica, 50(2), 127–138. 10.3161/00016454ao2015.50.2.001

[ece38782-bib-0002] Afdhal, B. , Charfi‐Cheikhrouha, F. , & Moali, A. (2013). Tunisian man‐made wetlands as alternative habitats for waterbirds and their role for conservation. African Journal of Ecology, 51(1), 154–163. 10.1111/aje.12022

[ece38782-bib-0003] Baker, A. J. , González, P. M. , Piersma, T. , Niles, L. J. , de Lima Serrano do Nascimento, I. , Atkinson, P. W. , Clark, N. A. , Minton, C. D. T. , Peck, M. K. , & Aarts, G. (2004). Rapid population decline in red knots: Fitness consequences of decreased refuelling rates and late arrival in Delaware Bay. Proceedings of the Royal Society of London. Series B: Biological Sciences, 271(1541), 875–882 10.1098/rspb.2003.2663 15255108 PMC1691665

[ece38782-bib-0004] Baschuk, M. S. , Koper, N. , Wrubleski, D. A. , & Goldsborough, G. (2012). Effects of water depth, cover and food resources on habitat use of marsh birds and waterfowl in boreal wetlands of Manitoba, Canada. Waterbirds, 35(1), 44–55. 10.1675/063.035.0105

[ece38782-bib-0005] Bellio, M. G. , Kingsford, R. T. , & Kotagama, S. W. (2009). Natural versus artificial wetlands and their waterbirds in Sri Lanka. Biological Conservation, 142(12), 3076–3085. 10.1016/j.biocon.2009.08.007

[ece38782-bib-0006] Bestelmeyer, B. T. , Ellison, A. M. , Fraser, W. R. , Gorman, K. B. , Holbrook, S. J. , Laney, C. M. , Ohman, M. D. , Peters, D. P. C. , Pillsbury, F. C. , Rassweiler, A. , Schmitt, R. J. , & Sharma, S. (2011). Analysis of abrupt transitions in ecological systems. Ecosphere, 2(12), 129. 10.1890/es11-00216.1

[ece38782-bib-0007] Cao, L. , Barter, M. , & Lei, G. (2008). New Anatidae population estimates for eastern China: Implications for current flyway estimates. Biological Conservation, 141(9), 2301–2309. 10.1016/j.biocon.2008.06.022

[ece38782-bib-0008] Cao, L. , Zhang, Y. , Barter, M. , & Lei, G. (2010). Anatidae in eastern China during the non‐breeding season: Geographical distributions and protection status. Biological Conservation, 143(3), 650–659. 10.1016/j.biocon.2009.12.001

[ece38782-bib-0009] Chen, B. , Cui, P. , Xu, H. G. , Lu, X. G. , Lei, J. C. , Wu, Y. , Shao, M. Q. , Ding, H. , Wu, J. , Cao, M. C. , & Liu, G. H. (2016). Assessing the suitability of habitat for wintering Siberian cranes (*Leucogeranus leucogeranus*) at different water levels in Poyang lake area, China. Polish Journal of Ecology, 64(1), 84–97. 10.3161/15052249pje2016.64.1.008

[ece38782-bib-0010] Cimprich, D. A. (2009). Effect of count duration on abundance estimates of Black‐capped Vireos. Journal of Field Ornithology, 80(1), 94–100. 10.1111/j.1557-9263.2008.00188.x

[ece38782-bib-0011] Collazo, J. A. , O'Hara, D. A. , & Kelly, C. A. (2002). Accessible habitat for shorebirds: Factors influencing its availability and conservation implications. Waterbirds, 25, 13–24.

[ece38782-bib-0012] Cong, P. H. , Cao, L. , Fox, A. D. , Barter, M. , Rees, E. C. , Jiang, Y. , Ji, W. , Zhu, W. , & Song, G. X. (2011). Changes in Tundra Swan Cygnus columbianus bewickii distribution and abundance in the Yangtze River floodplain. Bird Conservation International, 21(3), 260–265. 10.1017/s0959270911000098

[ece38782-bib-0013] Cong, P. H. , Wang, X. , Cao, L. , & Fox, A. D. (2012). Within‐winter shifts in lesser white‐fronted goose *Anser erythropus* distribution at East Dongting Lake, China. Ardea, 100(1), 5–11. 10.5253/078.100.0103

[ece38782-bib-0014] Cui, L. J. , Gao, C. J. , Zhao, X. S. , Ma, Q. F. , Zhang, M. Y. , Li, W. , Song, H. T. , Wang, Y. F. , Li, S. N. , & Zhang, Y. (2013). Dynamics of the lakes in the middle and lower reaches of the Yangtze River basin, China, since late nineteenth century. Environmental Monitoring and Assessment, 185(5), 4005–4018. 10.1007/s10661-012-2845-0 22965946

[ece38782-bib-0015] Dadaser‐Celik, F. , Bauer, M. E. , Brezonik, P. L. , & Stefan, H. G. (2008). Changes in the sultan marshes ecosystem (Turkey) in satellite images 1980–2003. Wetlands, 28(3), 852–865. 10.1672/07-182.1

[ece38782-bib-0016] Dakos, V. , & Bascompte, J. (2014). Critical slowing down as early warning for the onset of collapse in mutualistic communities. Proceedings of the National Academy of Sciences of the United States of America, 111(49), 17546–17551. 10.1073/pnas.1406326111 25422412 PMC4267327

[ece38782-bib-0017] Dang, Y. C. , He, H. S. , Zhao, D. D. , Sunde, M. , & Du, H. B. (2020). Quantifying the relative importance of climate change and human activities on selected wetland ecosystems in China. Sustainability, 12(3), 17. 10.3390/su12030912

[ece38782-bib-0018] Darnell, T. M. , & Smith, E. H. (2004). Avian use of natural and created salt marsh in Texas, USA. Waterbirds, 27(3), 355–361.

[ece38782-bib-0019] Diamond, J. M. (1969). Avifaunal equilibria and species turnover rates on the Channel Islands of California. Proceedings of the National Academy of Sciences of the United States of America, 64(1), 57–63. 10.1073/pnas.64.1.57 16591783 PMC286125

[ece38782-bib-0020] Du, Y. , Xue, H. P. , Wu, S. J. , Ling, F. , Xiao, F. , & Wei, X. H. (2011). Lake area changes in the middle Yangtze region of China over the 20th century. Journal of Environmental Management, 92(4), 1248–1255. 10.1016/j.jenvman.2010.12.007 21220184

[ece38782-bib-0021] Fan, J. , Wang, X. D. , Wu, W. , Chen, W. P. , Ma, Q. , & Ma, Z. J. (2021). Function of restored wetlands for waterbird conservation in the Yellow Sea coast. Science of the Total Environment, 756, 144061. 10.1016/j.scitotenv.2020.144061 33280877

[ece38782-bib-0022] Fang, J. , Wang, Z. H. , Zhao, S. Q. , Li, Y. K. , Tang, Z. Y. , Yu, D. , Ni, L. Y. , Liu, H. Z. , Xie, P. , Da, L. J. , Li, Z. Q. , & Zheng, C. Y. (2006). Biodiversity changes in the lakes of the central Yangtze. Frontiers in Ecology and the Environment, 4(7), 369–377.

[ece38782-bib-0023] Foti, R. , del Jesus, M. , Rinaldo, A. , & Rodriguez‐Iturbe, I. (2013). Signs of critical transition in the Everglades wetlands in response to climate and anthropogenic changes. Proceedings of the National Academy of Sciences of the United States of America, 110(16), 6296–6300. 10.1073/pnas.1302558110 23576751 PMC3631638

[ece38782-bib-0024] Fox, A. D. , Cao, L. , Zhang, Y. , Barter, M. , Zhao, M. J. , Meng, F. J. , & Wang, S. L. (2011). Declines in the tuber‐feeding waterbird guild at Shengjin Lake National Nature Reserve, China ‐ a barometer of submerged macrophyte collapse. Aquatic Conservation‐Marine and Freshwater Ecosystems, 21(1), 82–91. 10.1002/aqc.1154

[ece38782-bib-0025] Guan, L. , Lei, J. L. , Zuo, A. J. , Zhang, H. , Lei, G. C. , & Wen, L. (2016). Optimizing the timing of water level recession for conservation of wintering geese in Dongting Lake, China. Ecological Engineering, 88, 90–98. 10.1016/j.ecoleng.2015.12.009

[ece38782-bib-0026] Hallett, L. M. , Jones, S. K. , MacDonald, A. A. M. , Jones, M. B. , Flynn, D. F. B. , Ripplinger, J. , Slaughter, P. , Gries, C. , & Collins, S. L. (2016). Codyn: An R package of community dynamics metrics. Methods in Ecology and Evolution, 7(10), 1146–1151. 10.1111/2041-210x.12569

[ece38782-bib-0027] Hansson, L. A. , Bronmark, C. , Nilsson, P. A. , & Abjornsson, K. (2005). Conflicting demands on wetland ecosystem services: Nutrient retention, biodiversity or both? Freshwater Biology, 50(4), 705–714. 10.1111/j.1365-2427.2005.01352.x

[ece38782-bib-0028] Hou, J. J. , Li, L. , Wang, Y. F. , Wang, W. J. , Zhan, H. Y. , Dai, N. H. , & Lu, P. (2021). Influences of submerged plant collapse on diet composition, breadth, and overlap among four crane species at Poyang Lake, China. Frontiers in Zoology, 18(1), 10.1186/s12983-021-00411-2 PMC8130136 34001190

[ece38782-bib-0029] Hou, J. J. , Liu, Y. F. , Fraser, J. D. , Li, L. , Zhao, B. , Lan, Z. C. , Jin, J. F. , Liu, G. H. , Dai, N. H. , & Wang, W. J. (2020). Drivers of a habitat shift by critically endangered Siberian cranes: Evidence from long‐term data. Ecology and Evolution, 10(20), 11055–11068. 10.1002/ece3.6720 33144948 PMC7593143

[ece38782-bib-0030] Hou, X. J. , Feng, L. , Tang, J. , Song, X. P. , Liu, J. G. , Zhang, Y. L. , Wang, J. J. , Xu, Y. , Dai, Y. H. , Zheng, Y. , Zheng, C. M. , & Bryan, B. A. (2020). Anthropogenic transformation of Yangtze Plain freshwater lakes: Patterns, drivers and impacts. Remote Sensing of Environment, 248, 111998. 10.1016/j.rse.2020.111998

[ece38782-bib-0031] Hu, S. J. , Niu, Z. G. , Chen, Y. F. , Li, L. F. , & Zhang, H. Y. (2017). Global wetlands: Potential distribution, wetland loss, and status. Science of the Total Environment, 586, 319–327. 10.1016/j.scitotenv.2017.02.001 28190574

[ece38782-bib-0032] IUCN . (2021) The IUCN Red List of Threatened Species. Version 2021.3.www.iucnredlist.org

[ece38782-bib-0033] Jones, S. F. , Janousek, C. N. , Casazza, M. L. , Takekawa, J. Y. , & Thorne, K. M. (2021). Seasonal impoundment alters patterns of tidal wetland plant diversity across spatial scales. Ecosphere, 12(2), 3366. 10.1002/ecs2.3366

[ece38782-bib-0034] Kingsford, R. T. (1999). Aerial survey of waterbirds on wetlands as a measure of river and floodplain health. Freshwater Biology, 41(2), 425–438. 10.1046/j.1365-2427.1999.00440.x

[ece38782-bib-0035] Li, C. L. , Li, H. F. , Zhang, Y. , Zha, D. D. , Zhao, B. B. , Yang, S. , Zhang, B. W. , & de Boer, W. F. (2019). Predicting hydrological impacts of the Yangtze‐to‐Huaihe Water Diversion Project on habitat availability for wintering waterbirds at Caizi Lake. Journal of Environmental Management, 249, 109251. 10.1016/j.jenvman.2019.07.022 31401449

[ece38782-bib-0036] Li, Y. S. , Sleigh, A. C. , Ross, A. G. P. , Williams, G. M. , Tanner, M. , & McManus, D. P. (2000). Epidemiology of Schistosoma japonicum in China: morbidity and strategies for control in the Dongting Lake region. International Journal for Parasitology, 30(3), 273–281. 10.1016/s0020-7519(99)00201-5 10719120

[ece38782-bib-0037] Liang, J. , Meng, Q. F. , Li, X. , Yuan, Y. J. , Peng, Y. H. , Li, X. D. , Li, S. , Zhu, Z. Q. , & Yan, M. (2021). The influence of hydrological variables, climatic variables and food availability on Anatidae in interconnected river‐lake systems, the middle and lower reaches of the Yangtze River floodplain. Science of the Total Environment, 768, 144534. 10.1016/j.scitotenv.2020.144534 33454478

[ece38782-bib-0038] Liu, X. G. , Zhang, Q. , Li, Y. L. , Tan, Z. Q. , & Werner, A. D. (2020). Satellite image‐based investigation of the seasonal variations in the hydrological connectivity of a large floodplain (Poyang Lake, China). Journal of Hydrology, 585, 10.1016/j.jhydrol.2020.124810. 124810.

[ece38782-bib-0039] Ma, Z. J. , Cai, Y. T. , Li, B. , & Chen, J. K. (2010). Managing wetland habitats for waterbirds: an international perspective. Wetlands, 30(1), 15–27. 10.1007/s13157-009-0001-6

[ece38782-bib-0040] Ma, Z. J. , Wang, Y. , Gan, X. J. , Li, B. , Cai, Y. T. , & Chen, J. K. (2009). Waterbird population changes in the wetlands at Chongming Dongtan in the Yangtze River Estuary, China. Environmental Management, 43(6), 1187–1200. 10.1007/s00267-008-9247-7 19139954

[ece38782-bib-0041] Macarthur, R. H. , & Wilson, E. O. (1963). An equilibrium theory of insular zoogeography. Evolution, 17(4), 373–378. 10.2307/2407089

[ece38782-bib-0042] Monfils, M. J. , Brown, P. W. , Hayes, D. B. , Soulliere, G. J. , & Kafcas, E. N. (2014). Breeding bird use and wetland characteristics of dykes and undyked coastal marshes in Michigan. Journal of Wildlife Management, 78(1), 79–92. 10.1002/jwmg.637

[ece38782-bib-0043] Murkin, H. R. , Kaminski, R. M. , & Titman, R. D. (1982). Responses by dabbling ducks and aquatic invertebrates to an experimentally manipulated cattail marsh. Canadian Journal of Zoology, 60(10), 2324–2332. 10.1139/z82-299

[ece38782-bib-0044] Nakayama, T. , & Watanabe, M. (2008). Role of flood storage ability of lakes in the Changjiang River catchment. Global and Planetary Change, 63(1), 9–22. 10.1016/j.gloplacha.2008.04.002

[ece38782-bib-0045] Ntiamoa‐Baidu, Y. , Piersma, T. , Wiersma, P. , Poot, M. , Battley, P. , & Gordon, C. (1998). Water depth selection, daily feeding routines and diets of waterbirds in coastal lagoons in Ghana. Ibis, 140(1), 89–103. 10.1111/j.1474-919X.1998.tb04545.x

[ece38782-bib-0046] Ogden, J. C. , Baldwin, J. D. , Bass, O. L. , Browder, J. A. , Cook, M. I. , Frederick, P. C. , Frezza, P. E. , Galvez, R. A. , Hodgson, A. B. , Meyer, K. D. , Oberhofer, L. D. , Paul, A. F. , Fletcher, P. J. , Davis, S. M. , & Lorenz, J. J. (2014). Waterbirds as indicators of ecosystem health in the coastal marine habitats of Southern Florida: 2. Conceptual Ecological Models. Ecological Indicators, 44, 128–147. 10.1016/j.ecolind.2014.03.008

[ece38782-bib-0047] Olson, D. M. , & Dinerstein, E. (2002). The Global 200: Priority ecoregions for global conservation. Annals of the Missouri Botanical Garden, 89(2), 199–224. 10.2307/3298564

[ece38782-bib-0048] Palmer, M. , & Ruhi, A. (2019). Linkages between flow regime, biota, and ecosystem processes: Implications for river restoration. Science, 365(6459), 1264‐+. 10.1126/science.aaw2087 31604208

[ece38782-bib-0049] Paszkowski, C. A. , & Tonn, W. M. (2006). Foraging guilds of aquatic birds on productive boreal lakes: environmental relations and concordance patterns. Hydrobiologia, 567, 19–30. 10.1007/s10750-006-0053-z

[ece38782-bib-0050] Poysa, H. (1983). Resource utilization pattern and guild structure in a waterfowl community. Oikos, 40(2), 295–307. 10.2307/3544594

[ece38782-bib-0051] Poysa, H. , Holopainen, S. , Elmberg, J. , Gunnarsson, G. , Nummi, P. , & Sjoberg, K. (2019). Changes in species richness and composition of boreal waterbird communities: A comparison between two time periods 25 years apart. Scientific Reports, 9, 1725. 10.1038/s41598-018-38167-1 30741959 PMC6370776

[ece38782-bib-0052] Safran, R. J. , Isola, C. R. , Colwell, M. A. , & Williams, O. E. (1997). Benthic invertebrates at foraging locations of nine waterbird species in managed wetlands of the northern San Joaquin Valley, California. Wetlands, 17(3), 407–415. 10.1007/bf03161430

[ece38782-bib-0053] Scheffer, M. , & Carpenter, S. R. (2003). Catastrophic regime shifts in ecosystems: linking theory to observation. Trends in Ecology & Evolution, 18(12), 648–656. 10.1016/j.tree.2003.09.002

[ece38782-bib-0054] Scheffer, M. , Carpenter, S. , Foley, J. A. , Folke, C. , & Walker, B. (2001). Catastrophic shifts in ecosystems. Nature, 413(6856), 591–596. 10.1038/35098000 11595939

[ece38782-bib-0055] Sun, R. , Yao, P. P. , Wang, W. , Yue, B. , & Liu, G. (2017). Assessment of wetland ecosystem health in the Yangtze and Amazon River Basins. Isprs International Journal of Geo‐Information, 6(3), 81. 10.3390/ijgi6030081

[ece38782-bib-0056] Sun, Z. D. , Huang, Q. , Opp, C. , Hennig, T. , & Marold, U. (2012). Impacts and implications of major changes caused by the Three Gorges Dam in the middle reaches of the Yangtze River, China. Water Resources Management, 26(12), 3367–3378. 10.1007/s11269-012-0076-3

[ece38782-bib-0057] Tidwell, P. R. , Webb, E. B. , Vrtiska, M. P. , & Bishop, A. A. (2013). Diets and Food Selection of Female Mallards and Blue‐Winged Teal During Spring Migration. Journal of Fish and Wildlife Management, 4(1), 63–74. 10.3996/072012-JFWM-062

[ece38782-bib-0058] Wang, H. W. , Kuo, P. H. , & Dodd, A. E. (2020). Gate operation for habitat ‐oriented water management at Budai Salt Pan Wetland in Taiwan. Ecological Engineering, 148, 10.1016/j.ecoleng.2020.105761. 105761.

[ece38782-bib-0059] Wang, M. , Gu, Q. , Liu, G. H. , Shen, J. W. , & Tang, X. G. (2019). Hydrological condition constrains vegetation dynamics for wintering waterfowl in China's East Dongting Lake wetland. Sustainability, 11(18), 10.3390/su11184936

[ece38782-bib-0060] Wang, W. J. , Fraser, J. D. , & Chen, J. K. (2017). Wintering waterbirds in the middle and lower Yangtze River floodplain: changes in abundance and distribution. Bird Conservation International, 27(2), 167–186. 10.1017/s0959270915000398

[ece38782-bib-0061] Wang, X. , Fox, A. D. , Cong, P. H. , & Cao, L. (2013). Food constraints explain the restricted distribution of wintering Lesser White‐fronted Geese Anser erythropus in China. Ibis, 155(3), 576–592. 10.1111/ibi.12039

[ece38782-bib-0062] Weber, L. M. , & Haig, S. M. (1996). Shorebird use of South Carolina managed and natural coastal wetlands. Journal of Wildlife Management, 60(1), 73–82. 10.2307/3802042

[ece38782-bib-0063] Williamson, L. , Hudson, M. , O'Connell, M. , Davidson, N. , Young, R. , Amano, T. , & Szekely, T. (2013). Areas of high diversity for the world's inland‐breeding waterbirds. Biodiversity and Conservation, 22(6–7), 1501–1512. 10.1007/s10531-013-0488-2

[ece38782-bib-0064] Wu, H. P. , Chen, J. , Zeng, G. M. , Xu, J. J. , Sang, L. H. , Liu, Q. , Dai, J. , Xiong, W. P. , Yuan, Z. , Wang, Y. Q. , & Ye, S. J. (2020). Effects of early dry season on habitat suitability for migratory birds in China's two largest freshwater lake wetlands after the impoundment of Three Gorges Dam. Journal of Environmental Informatics, 36(2), 82–92. 10.3808/jei.201900411

[ece38782-bib-0065] Xia, S. X. , Wang, Y. Y. , Lei, G. , Liu, Y. , Lei, J. Y. , Yu, X. B. , Wen, L. , & Zhou, Y. M. (2017). Restriction of herbivorous waterbird distributions in the Middle and Lower Yangtze River Floodplain in view of hydrological isolation. Wetlands, 37(1), 79–88. 10.1007/s13157-016-0841-9

[ece38782-bib-0066] Xiang, Y. , Wang, S. , Zhang, Y. , & Dai, Z. (2021). Green development efficiency measurement and influencing factors of the paper industry in the Yangtze River Economic Belt. Water, 13(9), 10.3390/w13091286

[ece38782-bib-0067] Xie, C. , Huang, X. , Mu, H. Q. , & Yin, W. (2017). Impacts of land‐use changes on the lakes across the Yangtze floodplain in China. Environmental Science & Technology, 51(7), 3669–3677. 10.1021/acs.est.6b04260 28285517

[ece38782-bib-0068] Xu, M. , Dong, X. H. , Yang, X. D. , Chen, X. , Zhang, Q. H. , Liu, Q. , Wang, R. , Yao, M. , Davidson, T. A. , & Jeppesen, E. (2017). Recent sedimentation rates of shallow lakes in the middle and lower reaches of the Yangtze River: patterns, controlling factors and implications for lake management. Water, 9(8), 10.3390/w9080617

[ece38782-bib-0069] Yuan, Y. J. , Zeng, G. M. , Liang, J. , Li, X. D. , Li, Z. W. , Zhang, C. , Huang, L. , Lai, X. , Lu, L. , Wu, H. , & Yu, X. (2014). Effects of landscape structure, habitat and human disturbance on birds: A case study in East Dongting Lake wetland. Ecological Engineering, 67, 67–75. 10.1016/j.ecoleng.2014.03.012

[ece38782-bib-0070] Zedler, J. B. , & Kercher, S. (2005). Wetland resources: Status, trends, ecosystem services, and restorability. Annual Review of Environment and Resources, 30, 39–74. 10.1146/annurev.energy.30.050504.144248

[ece38782-bib-0071] Zhang, C. , Yuan, Y. , Zeng, G. , Liang, J. , Guo, S. , Huang, L. , Hua, S. , Wu, H. , Zhu, Y. , An, H. , & Zhang, L. (2016). Influence of hydrological regime and climatic factor on waterbird abundance in Dongting Lake Wetland, China: Implications for biological conservation. Ecological Engineering, 90, 473–481. 10.1016/j.ecoleng.2016.01.076

[ece38782-bib-0072] Zhang, M. , Ni, L. , Xu, J. , He, L. , Fu, H. , & Liu, Z. (2013). Annual dynamics of the wetland plants community in Poyang Lake in response to water‐level variations. Research of Environmental Sciences, 26(10), 1057–1063.

[ece38782-bib-0073] Zhang, P. Y. , Zou, Y. A. , Xie, Y. H. , Zhang, H. , Liu, X. K. , Gao, D. L. , & Yi, F. Y. (2018). Shifts in distribution of herbivorous geese relative to hydrological variation in East Dongting Lake wetland, China. Science of the Total Environment, 636, 30–38. 10.1016/j.scitotenv.2018.04.247 29702400

[ece38782-bib-0074] Zhang, P. Y. , Zou, Y. A. , Xie, Y. H. , Zhang, S. Q. , Chen, X. S. , Li, F. , Deng, Z. M. , Zhang, H. , & Tu, W. (2020). Hydrology‐driven responses of herbivorous geese in relation to changes in food quantity and quality. Ecology and Evolution, 10(12), 5281–5292. 10.1002/ece3.6272 32607151 PMC7319142

[ece38782-bib-0075] Zhang, P. Y. , Zou, Y. A. , Xie, Y. H. , Zhang, S. Q. , Zhu, F. , Chen, X. S. , Li, F. , Deng, Z. M. , Yao, Y. , & Song, Y. C. (2021). Phenological mismatch caused by water regime change may explain the population variation of the vulnerable lesser white‐fronted goose in east Dongting Lake, China. Ecological Indicators, 127, e107776. 10.1016/j.ecolind.2021.107776

[ece38782-bib-0076] Zhang, S. Q. , Zhang, P. Y. , Pan, B. H. , Zou, Y. A. , Xie, Y. H. , Zhu, F. , Chen, X. S. , Li, F. , Deng, Z. M. , Zhang, H. , & Yang, S. (2021). Wetland restoration in the East Dongting Lake effectively increased waterbird diversity by improving habitat quality. Global Ecology and Conservation, 27, e01535. 10.1016/j.gecco.2021.e01535

[ece38782-bib-0077] Zhao, M. J. , Cao, L. , & Fox, A. D. (2010). Distribution and diet of wintering Tundra Bean Geese Anser fabalis serrirostris at Shengjin Lake, Yangtze River floodplain, China. Wildfowl, 60, 52–63.

[ece38782-bib-0078] Zhao, M. , Cao, L. , Klaassen, M. , Zhang, Y. , & Fox, A. D. (2015). Avoiding competition? Site use, diet and foraging behaviours in two similarly sized geese wintering in China. Ardea, 103(1), 27–U108. 10.5253/arde.v103i1.a3

[ece38782-bib-0079] Zou, Y. A. , Tang, Y. , Xie, Y. H. , Zhao, Q. H. , & Zhang, H. (2017). Response of herbivorous geese to wintering habitat changes: conservation insights from long‐term population monitoring in the East Dongting Lake, China. Regional Environmental Change, 17(3), 879–888. 10.1007/s10113-016-1087-z

[ece38782-bib-0080] Zou, Y. A. , Zhang, P. Y. , Zhang, S. Q. , Chen, X. S. , Li, F. , Deng, Z. M. , Yang, S. , Zhang, H. , Li, F. Y. , & Xie, Y. H. (2019). Crucial sites and environmental variables for wintering migratory waterbird population distributions in the natural wetlands in East Dongting Lake, China. Science of the Total Environment, 655, 147–157. 10.1016/j.scitotenv.2018.11.185 30469060

