# Supplementary material for: Dyke demolition led to a sharp decline in waterbird diversity due to habitat quality reduction: A case study of Dongting Lake, China
Source: Ecol Evol. 2022 Mar 31;12(4):e8782. doi: 10.1002/ece3.8782 (PMC8969919; doi:10.1002/ece3.8782)
Supplement: Supplementary file 1 — Supplementary Material [file ECE3-12-e8782-s001.docx]

Appendix

Appendix S1. Summary of wintering waterbird surveys (2012/2013-2020/2021) in the dyke-demolished and preserved areas

| Conmon name | Scientific name | Dyke-demolished areas (DDA) | | | Dyke-preserved areas (DPA) | | |
| --- | --- | --- | --- | --- | --- | --- | --- |
|  |  | Recorded years (Before) | Recorded years (After) | Total individuals | Recorded years (Before) | Recorded years (After) | Total individuals |
| Siberian Crane | *Grus leucogeranus* (CR,Ⅰ) | 0 | 2 | 25 | 0 | 1 | 2 |
| Common Crane | *Grus grus* (Ⅱ) | 4 | 4 | 354 | 5 | 4 | 964 |
| Hooded Crane | *Grus monacha* (VU,Ⅰ) | 1 | 1 | 8 | 3 | 3 | 16 |
| Tundra Swan | *Cygnus columbianus* (Ⅱ) | 2 | 1 | 124 | 4 | 4 | 3980 |
| Red-crested Pochard | *Netta rufina* | 0 | 0 | 0 | 0 | 1 | 1 |
| Swan Goose | *Anser cygnoid* (VU,Ⅱ) | 4 | 1 | 148 | 2 | 1 | 12 |
| Bean Goose | *Anser fabalis* | 5 | 4 | 42510 | 5 | 4 | 76681 |
| Greater White-fronted Goose | *Anser albifrons* (Ⅱ) | 5 | 2 | 8985 | 5 | 3 | 515 |
| Lesser White-fronted Goose | *Anser erythropus* (VU,Ⅱ) | 5 | 3 | 24742 | 5 | 2 | 13710 |
| Greylag Goose | *Anser anser* | 1 | 0 | 17 | 3 | 2 | 420 |
| Bar-headed Goose | *Anser indicus* | 1 | 0 | 1 | 0 | 2 | 25 |
| Little Grebe | *Tachybaptus ruficollis* | 4 | 4 | 752 | 5 | 4 | 1052 |
| Great-crested Grebe | *Podiceps cristatus* | 2 | 0 | 58 | 5 | 4 | 473 |
| Smew | *Mergellus albellus* (Ⅱ) | 1 | 0 | 42 | 4 | 4 | 477 |
| Common Merganser | *Mergus merganser* | 0 | 1 | 12 | 1 | 1 | 97 |
| Great Cormorant | *Phalacrocorax carbo* | 4 | 2 | 888 | 5 | 4 | 8840 |
| Grey Heron | *Ardea cinerea* | 5 | 4 | 1823 | 5 | 4 | 1391 |
| Great Egret | *Ardea alba* | 5 | 4 | 383 | 5 | 4 | 584 |
| Intermediate Egret | *Ardea intermedia* | 1 | 1 | 3 | 0 | 0 | 0 |
| Little Egret | *Egretta garzetta* | 3 | 4 | 191 | 4 | 4 | 269 |
| Black-crowned Night-Heron | *Nycticorax nycticorax* | 0 | 0 | 0 | 0 | 1 | 1 |
| Eurasian Bittern | *Botaurus stellaris* | 2 | 0 | 5 | 0 | 0 | 0 |
| Black Stork | *Ciconia nigra* (Ⅰ) | 1 | 0 | 4 | 0 | 2 | 6 |
| Scaly-sided Merganser | *Mergus squamatus* (EN,Ⅰ) | 0 | 0 | 0 | 0 | 1 | 3 |
| Oriental Stork | *Ciconia boyciana* (EN,Ⅰ) | 1 | 0 | 1 | 0 | 2 | 4 |
| Dalmatian Pelican | *Pelecanus crispus* (NT,Ⅰ) | 0 | 0 | 0 | 1 | 0 | 2 |
| Siberian Gull | *Larus smithsonianus* | 4 | 1 | 62 | 5 | 4 | 602 |
| Caspian Tern | *Hydroprogne caspia* | 0 | 0 | 0 | 0 | 1 | 3 |
| Black-headed Gull | *Chroicocephalus ridibundus* | 1 | 1 | 46 | 5 | 4 | 1418 |
| Cattle Egret | *Bubulcus ibis* | 1 | 0 | 2 | 0 | 0 | 0 |
| Eurasian Spoonbill | ***Platalea leucorodia (Ⅱ)*** | 5 | 1 | 903 | 5 | 4 | 2461 |
| Pied Avocet | *Recurvirostra avosetta* | 1 | 0 | 1170 | 5 | 4 | 37882 |
| Northern Lapwing | ***Vanellus vanellus (NT)*** | 4 | 0 | 110 | 4 | 4 | 1611 |
| Grey-headed Lapwing | *Vanellus cinereus* | 1 | 0 | 768 | 0 | 0 | 0 |
| Grey Plover | *Pluvialis squatarola* | 1 | 0 | 240 | 2 | 0 | 557 |
| Kentish Plover | *Charadrius alexandrinus* | 2 | 0 | 111 | 2 | 1 | 65 |
| Swinhoe's Snipe | *Gallinago megala* | 0 | 0 | 0 | 0 | 1 | 1 |
| Common Snipe | *Gallinago gallinago* | 1 | 0 | 1 | 0 | 0 | 0 |
| Black-tailed Godwit | *Limosa limosa* (NT) | 1 | 0 | 90 | 3 | 1 | 157 |
| Eurasian Curlew | *Numenius arquata* (NT) | 0 | 0 | 0 | 3 | 2 | 61 |
| Spotted Redshank | *Tringa erythropus* | 5 | 2 | 818 | 4 | 4 | 597 |
| Common Greenshank | *Tringa nebularia* | 3 | 0 | 108 | 1 | 2 | 62 |
| Green Sandpiper | *Tringa ochropus* | 1 | 0 | 2 | 0 | 1 | 1 |
| Common Sandpiper | *Actitis hypoleucos* | 1 | 0 | 168 | 0 | 0 | 0 |
| Temminck's stint | *Calidris temminckii* | 0 | 0 | 0 | 1 | 0 | 4 |
| Dunlin | ***Calidris alpina*** | 5 | 1 | 8422 | 4 | 3 | 22922 |
| Common Coot | *Fulica atra* | 1 | 0 | 450 | 0 | 1 | 13 |
| Ruddy Shelduck | *Tadorna ferruginea* | 5 | 4 | 1798 | 5 | 4 | 1207 |
| Common Shelduck | *Tadorna tadorna* | 2 | 2 | 980 | 4 | 4 | 2071 |
| Eurasian Wigeon | *Mareca penelope* | 1 | 1 | 132 | 3 | 0 | 583 |
| Falcated Duck | *Mareca falcata* (NT) | 3 | 1 | 9074 | 5 | 4 | 108259 |
| Gadwall | *Mareca strepera* | 0 | 0 | 0 | 5 | 3 | 2261 |
| Eastern Curlew | *Numenius madagascariensis*（EN,Ⅱ) | 1 | 0 | 76 | 0 | 0 | 0 |
| Green-winged Teal | ***Anas crecca*** | 4 | 2 | 7294 | 5 | 4 | 27734 |
| Mallard | ***Anas platyrhynchos*** | 4 | 2 | 187 | 3 | 4 | 1796 |
| Eastern Spot-billed Duck | ***Anas zonorhyncha*** | 5 | 4 | 3904 | 5 | 4 | 2611 |
| Northern Pintail | *Anas acuta* | 1 | 0 | 500 | 1 | 1 | 113 |
| Northern Shoveler | *Spatula clypeata* | 2 | 0 | 6709 | 2 | 4 | 1158 |
| Ferruginous Duck | *Aythya nyroca* (NT) | 0 | 0 | 0 | 1 | 0 | 100 |
| Common Pochard | *Aythya ferina* (VU) | 0 | 0 | 0 | 1 | 0 | 1 |
| Baer's Pochard | *Aythya baeri* (CR,Ⅰ) | 0 | 0 | 0 | 1 | 0 | 4 |
| Tufted Duck | *Aythya fuligula* | 2 | 1 | 899 | 1 | 3 | 81 |

Notes:

The common and scientific names area based on Zheng (2015).

‘Before’ represents wintering seasons from 2013 to 2017 (Before dyke demolition); ‘After’ represents wintering seasons from 2018 to 2021 (After dyke demolition); ‘Total’ represents wintering seasons of the study periods (2015-2020).

Letters in parentheses indicate threatened waterbirds listed in the IUCN Red List (www.iucnredlist.org): CR, Critically Endangered; EN, Endangered; VU, Vulnerable; NT, Near Threatened. Ⅰand Ⅱ indicate threatened waterbirds listed in national levels Ⅰ and Ⅱ protected animals in China. N indicates the number of years when the species was recorded. Bold font in the table were species selected at the species level in this study.

**Appendix S2.** All Satellite images used in this study.

| No. | Date | Image Type | Path/Row | Water level in Chenglingji Hydrological station (m) |
| --- | --- | --- | --- | --- |
| 1 | 2011-3-4 | Landsat LT5 | 123/40 | 21.52 |
| 2 | 2011-6-8 | Landsat LT5 | 123/40 | 24.8 |
| 3 | 2011-7-26 | Landsat LT5 | 123/40 | 26.69 |
| 4 | 2013-4-26 | Landsat OLI8 | 123/40 | 23.95 |
| 5 | 2013-5-12 | Landsat OLI8 | 123/40 | 26.75 |
| 6 | 2013-5-28 | Landsat OLI8 | 123/40 | 28.29 |
| 7 | 2013-6-13 | Landsat OLI8 | 123/40 | 29.36 |
| 8 | 2013-7-31 | Landsat OLI8 | 123/40 | 29.29 |
| 9 | 2013-9-17 | Landsat OLI8 | 123/40 | 25.5 |
| 10 | 2013-12-6 | Landsat OLI8 | 123/40 | 20.62 |
| 11 | 2013-12-22 | Landsat OLI8 | 123/40 | 21.36 |
| 12 | 2014-1-23 | Landsat OLI8 | 123/40 | 21.11 |
| 13 | 2014-8-19 | Landsat OLI8 | 123/40 | 29.01 |
| 14 | 2014-10-6 | Landsat OLI8 | 123/40 | 28.12 |
| 15 | 2015-3-31 | Landsat OLI8 | 123/40 | 22.87 |
| 16 | 2015-4-15 | Landsat OLI8 | 123/40 | 24.81 |
| 17 | 2015-10-24 | Landsat OLI8 | 123/40 | 23.78 |
| 18 | 2015-11-25 | Landsat OLI8 | 123/40 | 25.66 |
| 19 | 2016-3-1 | Landsat OLI8 | 123/40 | 22.14 |
| 20 | 2016-7-23 | Landsat OLI8 | 123/40 | 33.17 |
| 21 | 2016-11-28 | Landsat OLI8 | 123/40 | 22.37 |
| 22 | 2017-2-16 | Landsat OLI8 | 123/40 | 20.73 |
| 23 | 2017-7-26 | Landsat OLI8 | 123/40 | 30.01 |
| 24 | 2017-10-30 | Landsat OLI8 | 123/40 | 27.18 |
| 25 | 2017-12-17 | Landsat OLI8 | 123/40 | 20.37 |
| 26 | 2018-2-3 | Landsat OLI8 | 123/40 | 22.72 |
| 27 | 2019-7-30 | Landsat OLI8 | 123/40 | 31.25 |
| 28 | 2019-10-18 | Landsat OLI8 | 123/40 | 23.83 |
| 29 | 2019-12-5 | Landsat OLI8 | 123/40 | 20.14 |
| 30 | 2020-2-9 | Landsat OLI8 | 123/40 | 22.7 |
| 31 | 2020-4-13 | Landsat OLI8 | 123/40 | 25.38 |
| 32 | 2020-4-29 | Landsat OLI8 | 123/40 | 23.3 |
| 33 | 2020-6-16 | Landsat OLI8 | 123/40 | 28.21 |
| 34 | 2020-8-3 | Landsat OLI8 | 123/40 | 33.97 |
| 35 | 2015-2-10 | Sentinel-1 |  | 20.67 |
| 36 | 2016-1-17 | Sentinel-1 |  | 23.18 |
| 37 | 2017-1-18 | Sentinel-1 |  | 22.02 |
| 38 | 2018-1-13 | Sentinel-1 |  | 21.44 |
| 39 | 2019-1-13 | Sentinel-1 |  | 23.72 |
| 40 | 2020-1-15 | Sentinel-1 |  | 21.33 |
| 41 | 2021-1-14 | Sentinel-1 |  | 22.43 |
| 42 | 2016-8-1 | Sentinel-2 | T49RFN | 32.16 |
| 43 | 2016-12-9 | Sentinel-2 | T49RFN | 21.28 |
| 44 | 2016-12-29 | Sentinel-2 | T49RFN | 21.05 |
| 45 | 2017-2-27 | Sentinel-2 | T49RFN | 21.48 |
| 46 | 2017-7-17 | Sentinel-2 | T49RFN | 31.73 |
| 47 | 2017-8-6 | Sentinel-2 | T49RFN | 26.92 |
| 48 | 2017-12-9 | Sentinel-2 | T49RFN | 21.21 |
| 49 | 2017-12-19 | Sentinel-2 | T49RFN | 20.38 |
| 50 | 2018-1-8 | Sentinel-2 | T49RFN | 20.79 |
| 51 | 2018-2-2 | Sentinel-2 | T49RFN | 22.72 |
| 52 | 2018-2-12 | Sentinel-2 | T49RFN | 21.58 |
| 53 | 2018-3-9 | Sentinel-2 | T49RFN | 21.46 |
| 54 | 2018-7-22 | Sentinel-2 | T49RFN | 31.08 |
| 55 | 2018-11-24 | Sentinel-2 | T49RFN | 25 |
| 56 | 2019-1-23 | Sentinel-2 | T49RFN | 23.18 |
| 57 | 2019-7-22 | Sentinel-2 | T49RFN | 31.92 |
| 58 | 2019-7-27 | Sentinel-2 | T49RFN | 31.54 |
| 59 | 2019-8-11 | Sentinel-2 | T49RFN | 30.35 |
| 60 | 2019-8-16 | Sentinel-2 | T49RFN | 29.56 |
| 61 | 2019-8-21 | Sentinel-2 | T49RFN | 28.27 |
| 62 | 2019-9-5 | Sentinel-2 | T49RFN | 25.21 |
| 63 | 2019-9-30 | Sentinel-2 | T49RFN | 24.52 |
| 64 | 2019-10-20 | Sentinel-2 | T49RFN | 23.45 |
| 65 | 2019-11-9 | Sentinel-2 | T49RFN | 23.14 |
| 66 | 2019-11-14 | Sentinel-2 | T49RFN | 22.45 |
| 67 | 2019-11-19 | Sentinel-2 | T49RFN | 21.21 |
| 68 | 2019-12-4 | Sentinel-2 | T49RFN | 20.08 |
| 69 | 2019-12-14 | Sentinel-2 | T49RFN | 20.39 |
| 70 | 2020-2-17 | Sentinel-2 | T49RFN | 23.02 |
| 71 | 2020-3-13 | Sentinel-2 | T49RFN | 24.08 |
| 72 | 2020-3-18 | Sentinel-2 | T49RFN | 23.91 |
| 73 | 2020-8-15 | Sentinel-2 | T49RFN | 32.5 |
| 74 | 2021-1-2 | Sentinel-2 | T49RFN | 21.33 |
| 75 | 2021-2-21 | Sentinel-2 | T49RFN | 20.92 |


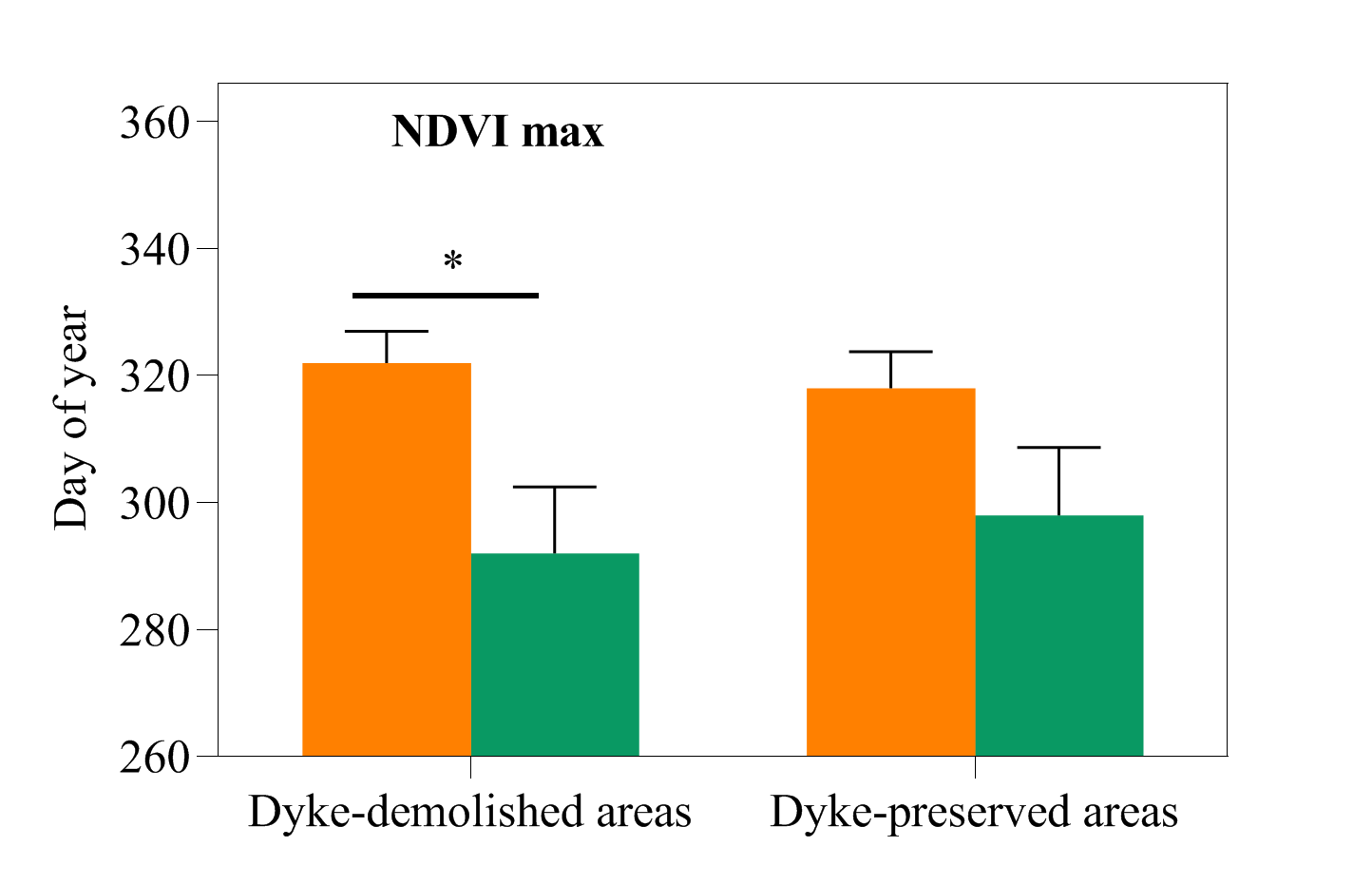


**Appendix S3.** Comparison of changes in NDVImax before and after dyke demolition. NDVImax represents the number of days when the vegetation reaches its maximum value in the first growing season. Error bars indicate the standard error of the mean. * denotes p < 0.05.
